# Face Masks to Combat Coronavirus (COVID-19)—Processing, Roles, Requirements, Efficacy, Risk and Sustainability

**DOI:** 10.3390/polym14071296

**Published:** 2022-03-23

**Authors:** Md Zillur Rahman, Md Enamul Hoque, Md Rubel Alam, Md Abdur Rouf, Saiful Islam Khan, Huaizhong Xu, Seeram Ramakrishna

**Affiliations:** 1 Department of Mechanical Engineering, Ahsanullah University of Science and Technology (AUST), Dhaka 1208, Bangladesh; 2 Department of Biomedical Engineering, Military Institute of Science and Technology (MIST), Dhaka 1216, Bangladesh; saifk28@gmail.com; 3 Department of Knitwear Manufacturing and Technology, BGMEA University of Fashion and Technology (BUFT), Dhaka 1230, Bangladesh; rubel@buft.edu.bd (M.R.A.); rouf_txt@buft.edu.bd (M.A.R.); 4 Department of Biobased Materials Science, Kyoto Institute of Technology (KIT), Matsugasaki Hashikamicho 1, Sakyoku, Kyoto 606-8585, Japan; 5 Department of Mechanical Engineering, National University of Singapore (NUS), Singapore 117575, Singapore; seeram@nus.edu.sg

**Keywords:** coronavirus, COVID-19, face mask, polymer, sustainability

## Abstract

Increasingly prevalent respiratory infectious diseases (e.g., COVID-19) have posed severe threats to public health. Viruses including coronavirus, influenza, and so on can cause respiratory infections. A pandemic may potentially emerge owing to the worldwide spread of the virus through persistent human-to-human transmission. However, transmission pathways may vary; respiratory droplets or airborne virus-carrying particles can have a key role in transmitting infections to humans. In conjunction with social distancing, hand cleanliness, and other preventative measures, the use of face masks is considered to be another scientific approach to combat ubiquitous coronavirus. Different types of face masks are produced using a range of materials (e.g., polypropylene, polyacrylonitrile, polycarbonate, polyurethane, polystyrene, polyester and polyethylene) and manufacturing techniques (woven, knitted, and non-woven) that provide different levels of protection to the users. However, the efficacy and proper disposal/management of the used face masks, particularly the ones made of non-biodegradable polymers, pose great environmental concerns. This review compiles the recent advancements of face masks, covering their requirements, materials and techniques used, efficacy, challenges, risks, and sustainability towards further enhancement of the quality and performance of face masks.

## 1. Introduction

Because of its great spreading potential, pathogenicity, and mortality, coronavirus (COVID-19) discovered on 31 December 2019, in Wuhan, China, has spread over the globe. COVID-19 is caused by SARS-CoV-2, a coronavirus that infects host cells through receptor-mediated endocytosis in conjunction with angiotensin-converting enzyme II (ACE2) [1]. Due to the extremely infectious characteristics of the COVID-19, WHO classified the health epidemic as a pandemic on 11 March 2020 [2]. Over 460,280,168 confirmed cases, including 6,050,018 fatalities [3] as of 16 March 2022, while the epidemic is still expanding. COVID-19 transmits from person to person through virus-carrying respiratory droplets expelled by infected individuals when they speak, cough, sneeze or exhale [4]. People nearby may inhale these droplets, and/or these can fall on bodies/surfaces that another person may touch, and subsequently become infected by touching their mouth, nose, and eyes [5,6]. SARS-CoV has a basic reproduction number of 3.28 (1.4 to 6.49), which is higher than WHO predictions of 1.4 to 2.5, meaning that an infected person can infect about 3 to 4 persons in a vulnerable population [6].

Fever, fatigue, myalgia, and dry cough are the most common symptoms of the COVID-19 [5,7,8,9]. Other referred symptoms are sore throat, chills, nausea, coryza, diarrhea, vomiting, sputum production, breath shortness, headache, arthralgia, nasal congestion, hemoptysis, and conjunctival congestion [6]. COVID-19 infection typically results in mild disease (i.e., mild pneumonia or non-pneumonia) in approximately 80% of cases, with the majority of patients recovering, while 14 % experience more serious disease and 6% suffer critical sickness [6,10,11,12]. Severe cases can result in severe pneumonia, cardiac injury, kidney failure, respiratory failure, and even death [5,6,10,11,13]. Pulmonary edema, septic shock, multiple organ dysfunction, and acute respiratory distress syndrome are all possible complications for critically ill patients [6,11].

Various public health measures, including self-isolation, quarantine [14], social distancing [9,15,16], lockdowns, and curfews, have been implemented by governments to limit the spread of coronavirus infection. Reduced population mobility and interpersonal interaction, school closures, reduced use of public transportation, visitor bans, and paid sick leave to keep infected workers at home may all assist in lowering disease transmissions [17]. Despite the fact that preventative measures have been implemented to curb the coronavirus spreading, it continues to have a significant effect on mental health owing to a variety of psychological, economic, and social factors, including social isolation, loneliness, stress, depression, anxiety, fear-induced over-reactive behavior, loss of loved ones, and loss of employment, and frustration, guilt, boredom, anger, sadness, nervousness, worry, helplessness, depression, and insomnia, [16,18]. Coronavirus has also created various types of societal stigma, including racism, discrimination, and judgmental behavior to isolated or quarantined people. Restrictions imposed on people during the coronavirus epidemic result in a substantial decline in mood, quality of life, and overall psychological well-being. In addition, the emergency has expanded into a worldwide public health and economic crisis affecting the $90 trillion global economies. Many people have lost their job due to the closure of a range of commercial sectors. However, public health interventions (e.g., quarantine) combined with limited mobility reduce carbon emissions, resulting in improved air quality and decreased water pollution in many places across the world [19,20,21,22]. This pandemic situation also highlighted the advantages of specific kinds of activities (e.g., pharmaceuticals and medicine) [23].

The most common and effective preventative strategies, however, require an improvement in cleanliness habits (e.g., reducing face touching, frequent hand hygiene, sanitization of surroundings, and use of tissues and other protectives). In most instances, these precautions were insufficient, and COVID-19 compelled individuals to alter their habits, such as masks wearing in public and avoiding physical contact, or maintaining social distance [24]. Using a face mask can effectively reduce transmissibility per contact by restricting the respiratory droplets of infected individuals. Public mask-wearing can be regarded as the most effective means of preventing the transmission of the COVID-19 when the compliance is high and proper guidelines are followed. Polypropylene and polyethylene are the commonly used plastic materials to fabricate the face mask. Other polymers such as polyethylene terephthalate, polycarbonate, polystyrene, polyvinyl chloride, polyamide, polyester, polylactic acid, and nylons are also used to manufacture them. However, improperly disposed of face masks made from long-lasting single-use plastics pose a new global environmental challenge. Therefore, this paper reviews the effects of face masks and their role in preventing coronavirus, materials and techniques used in manufacturing masks, associated challenges or risks involved in using masks, and their sustainability. Finally, the study concludes with concluding remarks.

## 2. Role of Face Mask in Combating COVID-19

Since Covid-19 disease mainly transmits through the spread of respiratory particles of infected individuals, reducing the rapid spread of the disease requires restricting direct contact with the infected individuals by ensuring quarantine (i.e., social distancing) and taking preventive measures to curb the transmission probability per contact [25]. According to the WHO, using a face mask alone is not enough to reduce the spreading or provide a reasonable level of protection against COVID-19; thus suggesting a face mask should be used as part of comprehensive preventive measures to curb the transmission and save lives [26]. The prevalence of evidence suggests that using a face mask effectively may reduce transmissibility per contact by restricting the respiratory particles of infected individuals in a community. When the compliance is high and proper guidelines are met, public mask-wearing is considered the most effective against the spread of the coronavirus. Several studies also indicated that the use of face masks by infected individuals is more effective than the susceptible people, i.e., health care providers in the context of community transmission to regional outbreaks. The evidence supporting the efficacy of public face mask-wearing can be divided into direct observational evidence and randomized controlled trials (RCTs), as discussed below.

### 2.1. Observational Evidence

There are only a few observational studies conducted in the case of the transmission of SARS-CoV-2; however, the efficacy of face masks in combatting COVID-19 is often backed by the observational evidence of face masks against other similar respiratory viruses (i.e., SARS). The evidence of using face masks against airborne transmission of respiratory diseases as a personal protective measure can be dated back to the 14th century, as stated by Wu [27]. Wu also identified cotton cloth face mask as the predominant means of personal protection, and his experiments showed that cotton cloth face mask is effective against airborne transmission of disease (i.e., Manchurian Plaque), as well as observational evidence of face mask efficacy for healthcare providers is present [28]. One study based on observational evidence conducted in Beijing households suggested that the use of face masks is found to be 79% effective against secondary transmission of SARS-CoV-2 when the compliance of every household member is high [29]. However, this study did not discuss the comparative efficacy and associated risks of different masks. 

A review study conducted by the Usher Institute suggested, based on epidemiological evidence, that mitigating aerosol dispersion and reducing the chances of transmission through respiratory droplets can be achieved even with homemade face masks used by infected people [30]. A systematic review study [31] concluded that the use of public face masking offers a significant result in reducing and preventing the spread of respiratory viruses, particularly in regional and global pandemic situations. However, the concrete benefit is limited by inconsistent adherence to the use of face masks by the general population. The study has supported its claims with epidemiological, theoretical, experimental, and clinical evidence from different studies. 

### 2.2. Randomized Control Trials

A study conducted by D. K. Chu et al. [32] indicated that using face masks in public places could reduce the risk of primary and secondary transmissions. They looked at three different preventive measures; maintaining social distancing, using face masks, and eye protection (i.e., using protective gear and avoiding direct touching) to reduce person-to-person transmission of SARS-CoV-2. The study reviewed only three studies of SARS transmission (not SARS-CoV-2) and the efficacy of using face masks outside healthcare settings. The control of the case study was not reliable enough to be applied in the case of SARS-CoV-2 transmission. This study also did not discuss the comparative efficacy and associated risks of different masks. However, the face mask is an effective personal protective tool that may reduce the risk of infection by 70% for an individual wearing a mask consistently while going public [33]. Considering 67 RCTs and observational evidence on physical interventions (i.e., face masks) to intercept the spread of respiratory disease viruses (e.g., SARS, not SARS-CoV-2), it was found that face masks are the most effective physical intervention across the mass population [34]. Another study evaluated face masks as an effective protective interception to enable ’source control’ for community outbreaks and protection for healthcare providers [35].

One RCT of face masks for influenza control in the general community in Australian households was conducted in 2008. The study found that when the compliance was high, the group of subjects with face masks had 80% more protective efficacy against influenza and similar diseases compared to the group without face masks [36]. Several other RCTs suggested that using face masks in combination with maintaining hand hygiene may effectively reduce the rapid spread of respiratory illnesses [37,38,39,40].

Overall, the evidence discussed above in terms of RCTs and observational studies supports the efficacy of using face masks; but is inconclusive. Also, the WHO and Cochrane both pointed out the lack of RCTs (due to logistical and ethical reasons) to investigate the impact and efficacy of face masks in community transmission in case of COVID-19 infection during this current pandemic [41]. As previously stated, the observational trials of the Beijing households [29] and Australian influenza RCT [36] found around 80% efficacy of face masks when the compliance is high among the subjects and about 70% efficacy for protecting the user himself. However, it is unclear if the findings mentioned for influenza and SARS can be applied similarly to SARS-CoV-2. Moreover, the number of observational studies conducted is inadequate for SARS-CoV-2, and the results may not be applicable universally around the globe. However, the use of public face masking by a healthy population possesses potential benefits against the community outbreaks, especially for COVID-19, where disease transmission may be rapid and pre-symptomatic. The use of face masks as ’source control’ is also effective and essential preventive measure during the COVID-19 pandemic.

## 3. Requirements and Types of Face Masks

**Figure 1:**
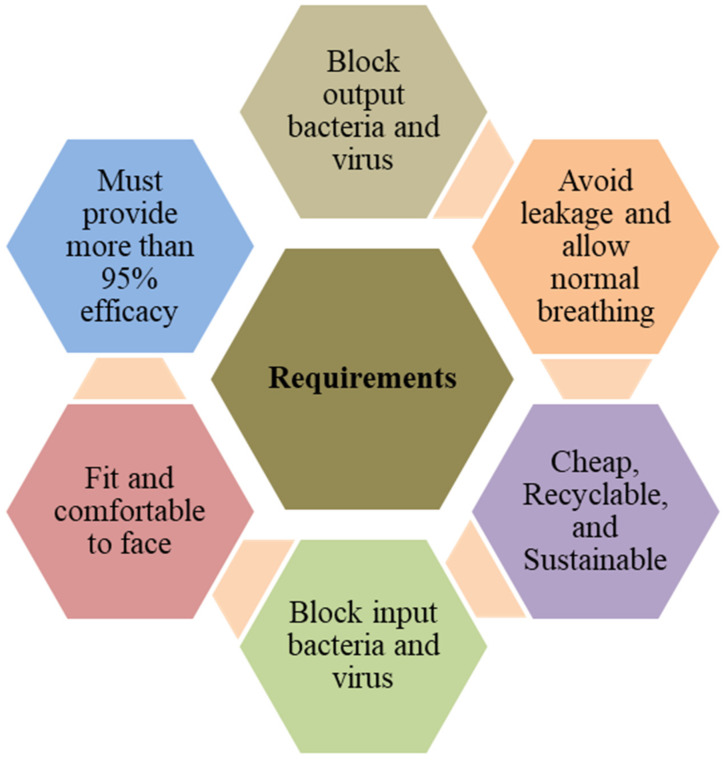
Major requirements of face masks.

There are different types of face masks (e.g., 3-ply surgical masks, a wide range of fabric masks, single-use face masks, and face shields) and respirators available commercially to protect individuals from getting and/or spreading COVID-19. Face masks are designed to protect primarily from respiratory droplets and particles to some extent. In contrast, the respirators are designed with extended protection against respiratory droplets, particles, and the virus that cause COVID-19. Both face masks and respirators have certain requirements to ensure performance and efficacy at a reliable and consistent level. Mask standards, ratings, and filtration effectiveness are presented in Table 1 National Institute for Occupational Safety and Health (NIOSH), ASTM International, and International Organization for Standardization (formerly known as International Association for Testing Materials) are engaged in creating an open and transparent consensus standards development process for addressing the standards gap identified during the wake of COVID-19 pandemic. The development of standards aimed to define performance requirements for source control, the protective efficacy of face mask or barrier face coverings, and standardized products to inform user selection decisions. The primary purpose of these specifications is to enable source control to protect the mass public with performance requirements such as protection capability, comfort, reusability, and so on. There are a few basic requirements (see Figure 1) of face masks to be used effectively, including an excellent fit over the nose and mouth of the user to prevent leaking (use of fitter, base, or nose wire) and the use of multi-layer non-woven but breathable fabrics for preventing bright light.

### 3.1. Medical Face Masks

Medical face masks, often referred to as disposable face masks or surgical masks (see the image in Table 2), are classified according to three levels of protection: level 1 (low), level 2 (moderate), and level 3 (high), with differing specifications (Table 2). Fit testing is not required for these masks, typically loosely fitted. They are frequently used in the healthcare setting with a low to moderate risk of acquiring airborne transmissible diseases [43]. Since there are regulatory restrictions, not all face masks qualify as surgical masks in the United States. The certification of the ASTM standards authority is mandatory for a mask to be qualified as a surgical mask. In Europe, European Standards Organization (EN) certifies surgical masks. Medical face masks must meet a few specifications for filtering according to the ASTM F2100-19e1 [44], including bacterial filtration efficiency (BFE), submicron particulate filtration efficiency (PFE), differential pressure (ΔP, an indicator of the breathability of the mask), flammability, etc. Both BFE and PFE must meet the requirements of ≥95% for low-barrier face masks (level 1) and ≥98% for moderate- and high-barrier face masks (levels 2 and 3). Surgical N95 respirators and other head/facial PPE (personal protective equipment) have flammability class 1, indicating relatively poor flammability. Compared to other surgical masks, Level 3 surgical masks offer the highest filtration efficacy, capable of filtering over 98% of particles of 3.0 μm in size, and have the highest possible fluid resistance [45]. On the contrary, in the European system, surgical masks are graded into type I-III, having the filtration capability same as their US counterparts. Surgical masks do not filter small particles effectively, although they can protect from directly spattering droplets. As surgical masks are loosely fitted around the face, they cannot prevent leaking from the lateral aspects of the masks [46]. Consequently, this mask is not recommended by the NIOSH, USA, to be used as particulate. However, surgical masks are capable of effectively blocking large respiratory droplets, splashes, and sprays during routine surgical or medical procedures.

### 3.2. Respirator Masks (N95 and FFP2 Variants)

**Figure 2:**
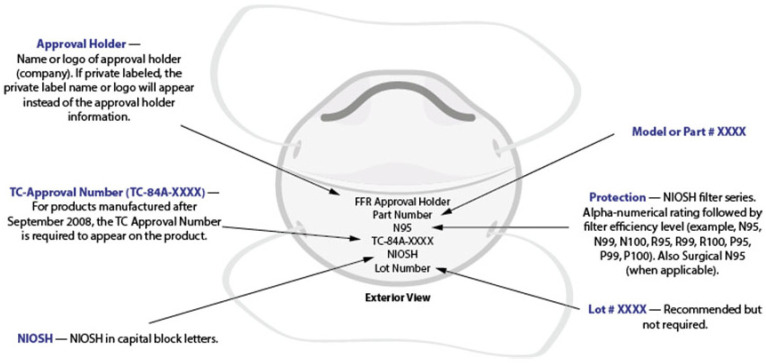
Model of a generic filtering respirator with appropriate markings [49].

The model of a generic filtering respirator with appropriate markings is illustrated in Figure 2. The respirators are designed, tested, and evaluated by NIOSH against a specific US standard, including quality requirements. The KN95 respirators are the most widely available and used respirators globally that meet standards at the international level. There are several other respirators of such standards; for example, DL2, DL3, DS2, DS3, FFP2, FFP3, KN100, KP95, KP100, P2, P3, PFF2, PFF3, and R95. An Emergency Temporary Standard (ETS) has been published by the Occupational Safety and Health Administration (OSHA) as the domestic demand and supply for respirators are increasing. As the Centers for Disease Control and Prevention (CDC) and NIOSH are committed to protecting health care workers as the foremost priority, the Food and Drug Administration (FDA) ensures that the health care provider should no longer use crisis capacity strategies. For such reasons, ETS was a necessary and timely step to address the COVID-19 crisis [47]. However, most commercially available KN95 respirators in different countries are counterfeit and do not comply with the NIOSH standard. For example, around 60% of KN95 respirators available commercially in the USA are fake. Therefore, CDC has published guidelines and standards to choose the right one for specific purposes. 

The respirators, class II medical devices regulated by the FDA, should be designed to ensure proper facial fit (i.e., designed to seal around the nose and mouth) and efficiently filter (up to 95%) airborne particles. The layering of the respirator may range between 3–5 layers of fabric and must pass the GB2626-2006 standard. Layer 1 is usually spun-bound made of non-woven cotton, which forms the shape and provides structural supports to the mask. The material should be excellent compatible with human skin. Layers 2 and 3 comprised of 2 filter layers (melt-blown fabric with a fiber diameter of 2 μm) should be capable of filtering dust, bacteria, and pollen. Spun-bound (non-woven cotton) or ES (ethylene-propylene side by side) hot hair cotton, a 2-component composite fiber, makes the layer 4. Layer 5 is made to give structural support and shape, and offers excellent compatibility and non-irritation to the skin of the user. However, the efficacy of the respirator depends on the weight of the material rather than the number of layers. Layers of melt-blown fabric somewhat provide a balance between filtering and breath resistivity. N95 (NIOSH-42CFR84 standard, United States), KN95 (GB2626-2006 standard, China), and FFP2 (EN 149-2001 standard, Europe) are not exactly similar, but they are certified as meeting the standards and can be expected to function very similarly to each other [48]. However, these masks are made for single use only and recommended to change after every patient encounter. After prolonged use, due to the tight-fitting and high filtering capacity, the wearers of respirator masks may experience discomfort and shortness of breath.

### 3.3. Single-Use Face Masks

Single-use face masks (see the image in Table 2) lack the requirements of surgical masks. Although their fabrication varies, single-use face masks are generally thin and made of only a single layer. If the surgical masks are abundant in supply, single-use face masks are not generally recommended to reuse in the healthcare setting. Although single-use face masks usually fail to filter tiny particles, they may still have the reasonably well capability to block the emission of large droplets and saliva. Additionally, in case of scarcity of the supply of surgical masks and respirator masks, single-use face masks can be an alternative to be used in the community setting, even for healthcare workers.

### 3.4. Cloth Face Mask

**Figure 3:**
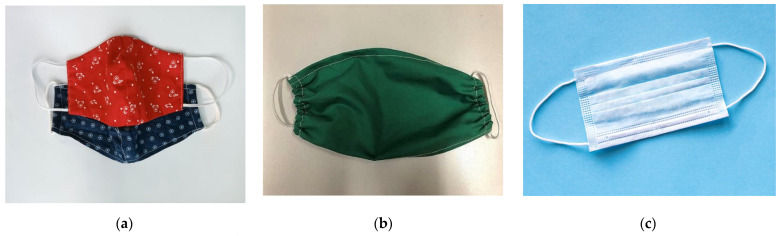

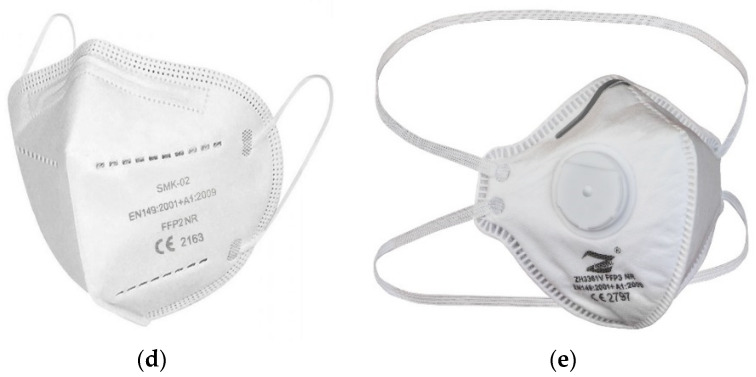
Various types of face masks: (**a**) cloth mask, (**b**) temporary fabric mask, (**c**) MNP mask or surgical mask, (**d**) FFP2 mask or N95 mask, and (**e**) FFP3 mask or respirator.

Cloth face masks (Figure 3a) made from various fabrics are widely available and are being used on a regular basis globally. However, NIOSH has recommended and evaluated such face masks according to the ASTM F3502-21 (standard for barrier face covering). NIOSH recommends wearing a face mask that meets ASTM F3502, ’workplace performance’, and ’workplace performance plus’. ASTM International also published F3050-17: Standard Guide for Conformity Assessment of Personal Protective Clothing and Equipment. The face masks should be able to filter at least 1 in every 5 nano-meter-sized particles from passing through the mask material and reaching the lungs of the user, which means a minimum sub-micron filtration score of 20% to qualify for the ASTM F3502 standard. It is also recommended that the user should either wear two masks combining a cloth mask and disposable mask or a 3-ply mask for better protection from COVID-19 [50]. 

Because of the severe scarcity of N95 respirators and surgical masks, several countries limit their usage by the general population, reserving them for individuals at the greatest risk of virus infection, such as healthcare professionals who come into contact with sick patients. The CDC, USA, released a recommendation in April 2020 encouraging the use of cloth face masks in public places to reduce the spread of COVID-19. This mask can be a reasonable solution when appropriate masks are in limited supply. Although cloth masks cannot protect against aerosols, they may limit viral transmission if other guidelines are followed, such as remaining at home, restricting unnecessary travel, and maintaining social distance.

The following kinds of masks are available in the context of SARS-CoV-2 [51]:

(1) Masks for daily usage (temporary fabric masks, Figure 3b): These masks do not protect against infection to the wearer. However, there is a slight risk decrease for droplet transmission, particularly during exhale, which minimizes the possibility of viral spreading. Although these masks are widely suggested for the general public while strolling, shopping, or using public transport, these masks should not be worn in the medical setting.
(2) MNP (medical mouth–nose protection; see Figure 3c): It is also known as a "surgical mask." For preventing infection, strict restrictions govern the industrial manufacture of MNP. The filtering performance is comparable to that of daily use masks, and they are designed to protect patients. They have been authorized for use by medical personnel, and their main purpose is to protect patients from aerosols.
(3) FFP2 mask (face filtering component, Figure 3d)/N95-mask: FFP2 masks meet stricter safety standards. They shield the wearer’s lungs by preventing more than 95% of particles and droplets from entering the lungs during breathing. As long as there is no exhaling valve, FFP2 masks efficiently protect the environment. In contrast, masks with an exhaling valve enable exhaled air to escape unfiltered, contaminating the environment.
(4) FFP3 mask: FFP3 masks (Figure 3e) protect the user even better than FFP2 because they filter out more than 99% of droplets and particles when inhaled. In the absence of an exhaling valve, FFP3-masks also protect the environment.

## 4. Materials and Methods Used in Manufacturing Face Masks

### 4.1. Mask Materials

Face mask materials are typically synthetic thermoplastic polymers with smoother morphology, uniform nanopore structure and size, low cost, and good bonding ability. Although polymers are widely used, they have some shortcomings, such as lack of good viral filtration efficiency, bacterial filtration efficiency, particulate filtration efficiency, and breathing comfort due to increased breathing resistance. So polymer blends of natural and synthetic additives are applied to increase the performance, efficiency, and safety of air filters and masks [52]. Chitosan, alginate, collagen, gelatin, silk fibroin, keratin, prolamin-based protein, silver nanoparticles, and natural extracts are also used to fabricate face masks. Commonly used mask materials and their products and properties are presented in Table 3. Nylon is suitable for producing face masks due to its high affinity for particulates and adequate air permeability, but it may create thermal discomfort. To address this issue, nylon can be combined with nanoporous PE, which has sufficient cooling capabilities, minimal pressure drops, and a filtration efficiency of 99.6% at high temperatures. N95 mask filters are made of nylon, polyester, polypropylene (PP), and cotton [53]. The filtration efficiency of polyester and nylon is in the range of 5–25%, and PP has a filtration efficiency of 6–10%, but it can be increased by triboelectrically charging [45]. Because of massive hydroxyl bonds, the PVA electrospun layer has recently replaced the melt-blown PP inner layer [54]. However, PP (i.e., plastic) shows some drawbacks, such as CO_2_ emissions during its production and PP’s inability to withstand high heat [55,56,57,58,59,60,61,62,63,64,65]. Water resistance is one of the requirements of a face mask. Polytetrafluoroethylene (PTFE) has been the most commonly used polymer for manufacturing waterproof membranes; however, the main issues are high cost and membrane porosity. Amini et al. [66] used a polyvinylidene fluoride electrospun membrane and a hydrogel electrospun mat to create a waterproof breathable membrane for face masks, which is a superior alternative to PTFE. High filtration efficiency and hydrophobicity can be achieved through the fiber deposition of cellulose acetate, whereas Polyacrylonitrile (PAN) and graphene oxide/gelatin demonstrate potential as face mask filters [45]. Low surface energy polymers such as PP, PE, PET, and polyvinylidene chloride can be used to create hydrophobic membranes [67]. Natural antibacterial polymers or extracts are also used in solution electrospinning along with prescribed polymers to obtain excellent performance.

### 4.2. Mask Manufacturing Techniques

**Figure 4:**
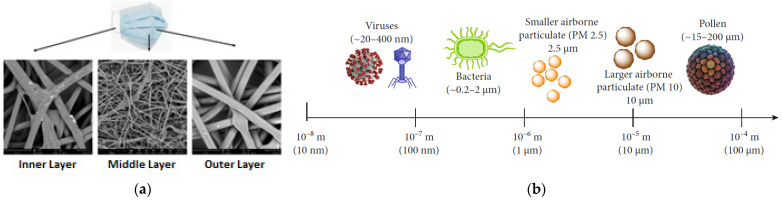
(**a**) SEM images of a surgical face mask [79] and (**b**) Sizes of common airborne contaminants and pathogens [71].

Surgical face masks can be made using three fabrication techniques: woven, knitted, and non-woven, but non-woven is the most convenient and cost-effective, with additional filtration efficacy [68]. Figure 4a demonstrates the SEM images of spunbond-meltblown-spunbond (SMS) of a surgical face mask, while the sizes of common airborne contaminants and pathogens are shown in Figure 4b. In a mask, non-woven SMS fabrics are typically used, which are spun-bond PP fabric (absorbent), melt-blown PP interlayer (electrically charged small fibrous material with random orientation), and spun-bond PP (blue color and hydrophobic) fabric on the outside [67,69,70,71]. Spunbond–meltblown–meltblown–spunbond (SMMS), spunbond–meltblown (SM), spunbond–spunbond (SS), spunbond–spunbond–spunbond (SSS), and spunbond–spunbond–meltblown–meltblown–spunbond (SSMMS) are the other most prevalent combinations of structures in various weight ranges [72]. Compared to other two layers, the middle layer contains fewer voids and acts as a filter, preventing harmful particles from entering [43,73]. The inner non-filtration non-woven is comfortable and soft, and absorbs fluids generated by breathing, coughing, and spitting, whereas the outer layer is hydrophobic. The PP, PLA, PET, PE, PAN, polyester, polyolefin, thermoplastic, natural cotton, regenerated rayon, cellulose acetate, or their mixing-blending fibers make the spun-bond layer [45,67,74]. The fabrication method, web structure, and cross-sectional shape of the fiber affects the surgical face mask filtration efficiency [75]. 

Fibers are hydroentangled, needle punched, thermally or chemically processed to make 20–35 gsm fabric [75]. The melt-blown intermediate hydrophobic porous structure is made of PP fibers; however, polyester derivatives are sometimes employed instead. The thermoplastic fiber diameter ranges between 0.1 and 10 microns to achieve high filtration efficiency, high strength, lightness, and good breathability [44]. High-porosity spun-bond fabrics have a thickness of 100 to 1000 microns and a larger fiber diameter (15–40 microns) than melt-blown fabrics [45,76]. Porosity and fiber uniformity increase as fiber diameter reduces [77]. For adults, children, and newborns, surgical face masks are 17.5 cm × 9.5 cm, 14.5 cm × 9.5 cm, and 12 cm × 7 cm in size, respectively.

Since coronavirus size varies between 60–140 nm or 0.06–0.14 microns, the surgical mask is ineffective against the coronavirus, but it has good resistance to a different strain of other viruses. The pore size of SMS fabric materials ranges from 11 to 44 µm [78].

**Figure 5:**
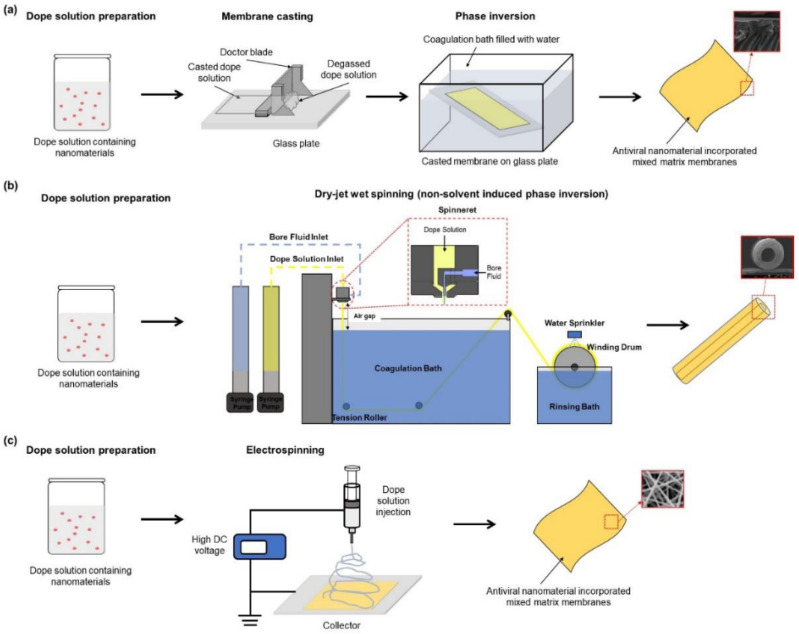
Three types of phase inversion: (**a**) dry-jet wet spinning, (**b**) non-solvent-induced phase separation, and (**c**) electrospinning [84].

Electrospun nanofibrous membranes (0.04–2 micron) have high potential for various applications such as tissue engineering [80,81,82] and water/air filtration [83], which can be used in place of melt-blown non-woven for high filtration efficacy [45,73]. The use of nanomaterials (silver (Ag), gold (Au), copper (Cu), titanium (Ti), zinc oxide (ZnO), and carbon (C)) in the nanofibrous membrane can make it more effective against waterborne and airborne bacteria/viruses [84]. Ultrasonic welding is used to join the layers together. The sharp edges of GO (graphene oxide) and rGO (reduced graphene oxide) nanosheets destroy the viral envelope and spikes. The nanomaterials membrane fabrication methods are shown in Figure 5.

**Figure 6:**
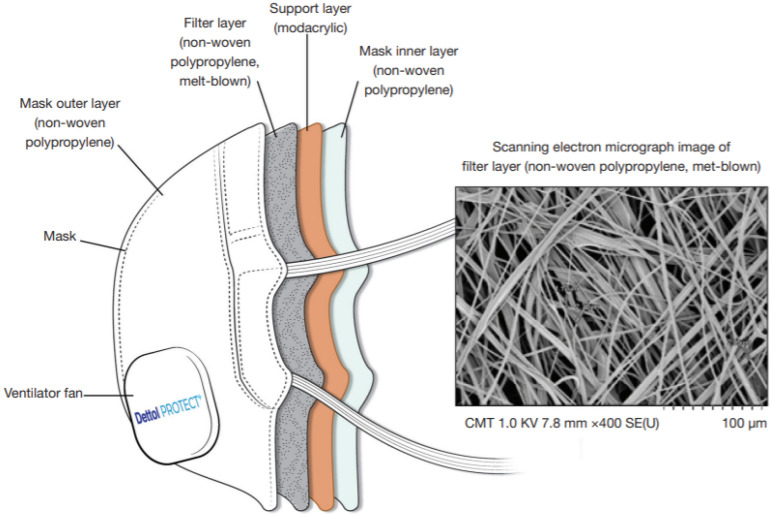
Four-layer N95 respirator mask [67].

The major differences between the three methods are fiber size, strength, and permeability. Due to the reduced filtration efficiency of this type of face mask, the N95 mask is developed to resist more fine particles (less than 5 microns). Virus spread can be prevented by wearing a four-layer N95 face mask (see Figure 6). It has a spun-bond PP outer layer, a cellulose/polyester second layer of filter material, a melt-blown PP third layer of filter material, and a spun-bond PP fourth layer of the inner layer [57]. The surgical mask weighs 3.5 g, while the N95 mask weighs 18.14 g. Recently, a good face mask process based on antiviral and antibacterial nanotechnology has been under investigation. Alternatively, a needle punched 4-layer face mask can provide acute protection by embedding positively charged Ag, Mg, Cu, ZnO, Ti, GO, and zeolite nanomaterials in the outer two layers while enhancing antibacterial nanoparticles in the middle two layers. However, most researchers found the adverse effects of nanoparticles (NPs) on human respiration, bone marrow, lymph nodes, the spleen, the brain, the liver, the heart, and the lungs. For example, nano-silver is toxic to mammalian liver cells, stem cells, and even brain cells. Furthermore, nano-silver may adversely impact the ability to control harmful bacteria by increasing antibiotic resistance, which may have a negative impact on human health [85].

**Figure 7:**
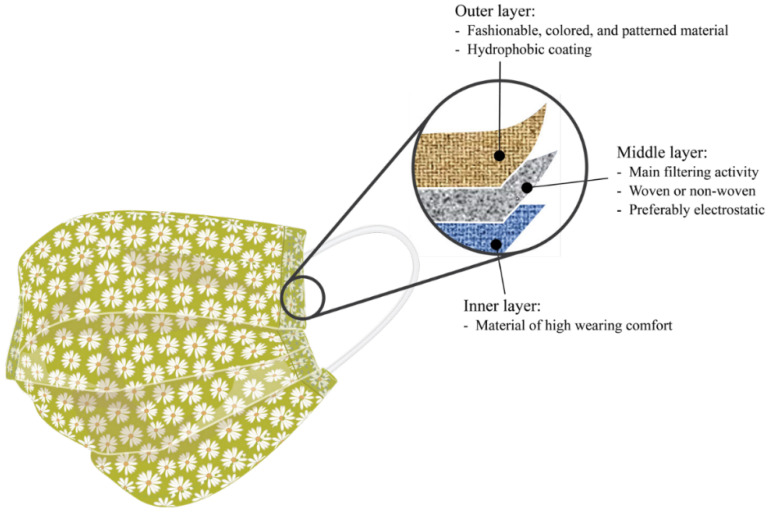
Cloth masks material features [86].

In some cases, a high-mechanical-strength fibrous composite is used in the face mask. Due to a lack of commercial face masks to counteract COVID-19, household and clothing fabrics are chosen as face mask materials [71]. The use of household masks is much more ambiguous and offers less effectiveness (39–65%). Such masks are currently being used in large numbers due to the sudden increase in demand and their ability to reduce exposure [68]. Fabrics such as cotton and silk, as well as kitchen towels and pillowcases, are typically used and sewed together in a short amount of time. A new reusable three-layer coated face mask was developed with good filtration for a high life span, as illustrated in Figure 7 [86]. Wang et al. [29] studied seventeen homemade fabric masks and found that their combinations met several surgical mask standards. Many factors influence the performance of a homemade mask, including the mask material and manufacturing technique. Pathogen particles, speed, and the ability to sit pleasantly against the wearer’s face are all factors that need to be considered. However, the efficacy of fabric masks depends on fabric sett, texture, and structure. Another recent mask engineering in improving the filter fabrics effectiveness was discussed by Chua et al. [71]. The best way to construct electret membranes is to use charged electrostatic processes (in situ charging, corona charging, and tribo charging) [87]. The efficiency of highly porous crystalline nanoparticles alone and in combination is equally promising [71]. Furthermore, the viral deactivation ability of bio-based antibacterial extracts embedded in the nanofibrous mat is excellent [88]. 

## 5. Efficacy of Currently Available Face Masks

A face mask prevents the viral transmission from inhalation or/and exhalation as a mouth and nose covering (MNC) in public places. To control the outbreak, we must wear the mask to reduce the spread of pathogen-containing aerosols and liquid droplets in our daily lives or from a medical perspective. MNCs and self-made masks are not “leak-proof” and do not provide total respiratory protection because air can escape through them. The WHO recommends wearing a face mask to prevent the spread of the coronavirus. However, the effectiveness of a face mask in preventing aerosols and droplets is unknown. Face masks with porosity less than 0.06 microns effectively clog the coronavirus, ranging in size from 0.06 to 0.14 microns. Many shapes and materials are available from face mask manufacturers. Combating the spread of fast-moving infectious diseases has long been practiced with cotton masks, surgical masks, and N95 respirator masks. Wearers emit nearly 20,000 droplets per second, ranging from 20 to 500 um in size. SARS-CoV-2, SARS, and MERS infections can be significantly reduced with N-95, surgical, or similar multilayer cotton masks. The clinical efficacy of a mask is influenced by factors such as the filtering ability and compliance with wearer instructions [89]. The effectiveness of face masks is mainly determined by the filtration efficacy (fabric material) and facepiece leakage (fit) [67,90]. In addition to the material, the masks’ fit can also be taken into consideration (Greig et al., 2021). A tight fit and good seal at the nose and cheeks are the most important considerations for fitting the face with a mask. If the filter is not properly installed, it will not perform optimally [91]. As a result, both thermoplastic layering qualities and functionalized materials must be considered in the design of the face mask’s construction [92]. The face mask respirators N95, R95, and P95, have efficacies of almost 95, 99, and 99.97%, respectively, in the United States [67]. However, cough aerosol is blocked by 59, 51, 47, and 60% by surgical masks, three-layer cotton cloth masks, single-layer polyester masks, and double-layer polyester masks, respectively [93,94,95]. Surgical masks require a current average BFE of 95% [71]. The efficiency of cloth masks varies depending on the type and structure of the fabric [46,96,97,98]. The sewn mask with a vacuum cleaner filter shows more noticeable results (70%) than a typical cloth mask [91]. The more the layer, the more the filtration efficiency but less breathing comfort. Results suggested using two to four layers to secure both benefits [99]. Filtering facepieces (FFP), half masks, powered air-purifying respirators (PAPR), and SAR (atmosphere-supplying respirators) are available in Europe. There are three classes of FFP, with respective efficiencies of 80%, 94%, and 99%. It has the maximum filtration efficiency and meets N95 standards [69,100,101]. 

Many types of cotton knitted fabrics and towels can be used as mask materials to prevent transmission, even though they are less effective than surgical masks and N95 respirator masks. Studies showed that different metal oxides fibrous layers on mask material improve the effectiveness [102]. Masks with activated carbon or HVAC air filters demonstrate good results [103]. Fabric masks appear less effective than surgical masks and N95 respirators [69]. In rare cases, cloth masks are recommended to suppress an epidemic in a low-population setting when other masks are inaccessible [89]. Fabric masks are rarely used in medical settings because surgical masks and respirators are widely available. As opposed to surgical masks, respirators keep small aerosol particles out of the body. Humans are moderately protected from viruses by wearing a cloth face mask [104]. People in Bangladesh typically wear cloth masks to protect themselves from dust particles. Healthy people were also tested for their ability to decrease aerosol particle emission rates from breathing, speaking, and coughing using unvented KN95 respirators, vented N95 respirators, surgical masks, and homemade paper and cloth masks. Surgery masks, KN95 respirators without vents, and N95 respirators with vents significantly reduce the number of particles emitted. However, surgical masks have three times better performance than homemade masks [89,104]. The surgical face mask shows better efficiency than other forms, and 70% ethanol treatment affects the efficiency [75]. Apart from that, the N95 respirators mask protects better against germs and viruses among surgical medical masks [90]. Other spatial extraneous factors, such as respective distance and moist weather, affect the efficacy [105]. 

The effectiveness of face masks has been the subject of very few studies. Fabric masks were found to be less effective than surgical masks and N95 respirators. Filtration efficiency ranges from 15 to 57% for three particle sizes (30, 100, and 500 nm) [68]. As an alternative, a four-layer cotton mask may provide similar benefits. However, the efficacy can be improved by 6–10% with a simple triboelectrically charge [76]. This can be especially effective in case of severe infection. A surgical mask can be worn inside and a cotton mask outside to combat the virus. The effectiveness of a homemade mask was almost 95% in another study, which used a single layer of nylon fabric sandwiched between four layers of kitchen filter. A negative ion emitter should be used in specified conditions to improve the filtration efficiency of medical masks [69]. Notably, the face shield is 10 times more effective than the standard face mask [106]. According to the literature, frequent handwashing, wearing a face mask, gloves, and gowns together can limit transmission by 91%, although individually they can reduce the transmission by 55%, 68% (N95-91), 57%, and 77%, respectively [107]. N95 protected against influenza and SARS virus in 2018 [108,109], while N95 offered protection against influenza and rhinovirus in 2020 [46]. Cotton masks also provide good protection. Different mask materials have different efficacies, as presented in Table 4.

## 6. Challenges and Risks in Using Face Masks

Face masks pose risks and challenges such as uncertain efficacy, cost, fit, availability, and so on. Moreover, the lack of interest among rural residents in wearing masks in public gatherings may represent a severe problem. Surgical and cloth masks provide less external protection because of their weaker barrier capability. When the user releases pressurized droplets or aerosolized particles (e.g., during a cough or sneeze), these particles are more likely to escape from the sides than the front due to the fitting. Cloth masks are open to allow air from the sides [45]. The mask should be airtight to the wearer to have a decent outcome. Aside from that, various studies highlight the primary arguments in favor of wearing a face mask, such as a false sense of security (protects only those in the immediate vicinity), proper use, and incorrect communication. Long-term usage, reuse, and decontamination are among the issues of face masks noted by researchers worldwide. Because of the exposure time, the N95 mask might be worn for up to 8 h [110]. If a proper disinfection approach is followed, it is possible to reuse after use. Wearing a face mask over a respirator is also recommended for easier cleaning and disposal. Another important difficulty and concern is a supply constraint compared to global demand [68]. It is essential to supply face masks to a great extent to combat the corona effects. Another challenge in developing countries is the availability of the appropriate mask [111]. Due to a scarcity of disposable commercial face masks, the nation will eventually shift to local production [111]. For meeting the instant demand, some unauthorized industries may manufacture masks that compromise quality. 

The use of synthetic polymer has raised some environmental threats, such as non-renewable and non-biodegradable [72]. In the case of the PP face mask, COVID-19 has a detrimental effect on one’s health and finances and has an adverse impact on the environment. A vast amount of spun-bond or melt-bond PP sheets are manufactured every year worldwide. Because of the frequent use of face masks, a substantial amount of infectious waste is inevitably generated [111]. However, selecting appropriate fabrics, reusing, and extended use are still challenges [110]. As a result, reusability is in high demand for two reasons. Not all masks are reusable (disposable masks). Face masks should not be used repeatedly (maximum two times, depending on types [111]); if this is the case, the area must be disinfected using a safe chemical. Poor people usually do not want to spend money on masks since lifesaving needs take precedence.

**Figure 8:**
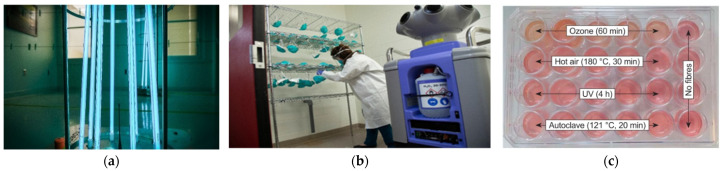
(**a**) UV radiation to N95 mask at University of Nebraska and (**b**) Hydrogen peroxide vapor generator at Duke University Hospital [118], and (**c**) Sterilization conditions [117].

**Figure 9:**
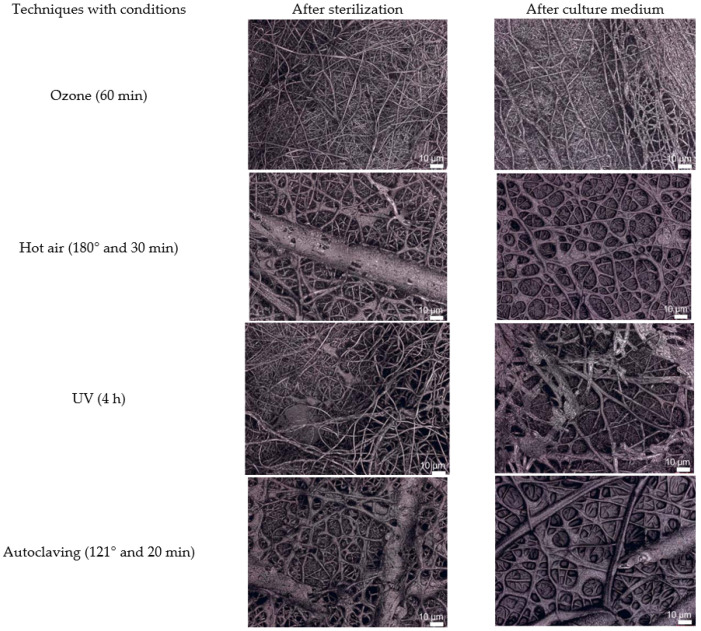
CLSM images of nanofibrous mats captured after sterilization and immersion in the culture medium [117].

To make face masks mandatory for everyone, the government must provide enough masks and support those who cannot afford them, such as the poor and frontline health professionals. People are typically willing to wear a less effective cotton mask for long periods without washing it. Furthermore, they lack knowledge of how to disinfect masks. Despite wearing a mask, a man touching his nose or mouth repeatedly (23–26 times per hour) with an unsensitized hand is a serious worry. Several disinfection processes are proposed after the researchers have inserted a proper electrostatic charge, including irradiation, fumigation, ethylene oxide, ozone, boiling in hot water, and steaming [112,113,114,115,116]. Some disinfection processes are shown in Figure 8a,b. Furthermore, the nanofibrous mat can be sterilized effectively using autoclaving, UV sterilization, ozone treatment, heat sterilization, or gamma radiation (Figure 8c). The results showed the morphology after sterilization and cell culture of a PAN/gelatin mat, and it was suggested that both hot air and autoclaving change the morphology, whereas ozone treatment changes the chemistry of gelatin. Figure 9 depicts confocal laser scanning microscopy (CLSM) images of sterilization, fiber morphology, and cell culture [117].

There are no significant differences between new and sterilized masks, and all methods are acceptable because it is a health issue [112]. Using 70% ethanol severely affects the performance of the face mask [94]. The mask can be used for a more extended period after being exposed to UV radiation and peroxide fumes for 15–20 mins [68,71,116]. Sterilizing the mask with steam for two hours and hot water at 800 °C for ten minutes is effective without compromising its efficacy. Avian coronavirus can be inactivated in just five minutes of steam exposure.

After immediate use, the mask should be disposed of properly as infectious waste. In developing countries, there are no facilities to dispose of these wastes. The risk of coronavirus infection increases after spending several days in an open field with solid municipal wastes. Besides waste pickers and street vendors, children and others who have easy access to face masks that may contain the virus are also at risk of contracting the disease. A coronavirus infection can be spread through masks, gloves, and tissues, among other items. Therefore, COVID-19 prevention efforts can be compromised by a lack of management and guidance. General characteristics of different types of masks and suggestions to combat COVID-19 are presented in Table 5.

## 7. Sustainability of Face Masks in Combatting COVID-19

Cellular and acellular microorganisms in respiratory aerosols have played an important role in disease transmission and communal to global outbreaks in the past century, especially with the COVID-19 pandemic. Surgical face masks, non-surgical face masks, and respirators have been used by healthcare providers, patients, and the general population as a basic PPE device to reduce transmission. This has triggered a sudden indiscriminate spike in demand in the manufacturing industry and more than four million tons of plastic waste daily [72]. Both ways resulted in an unsustainable system with ever-growing challenges in waste management with added environmental and economic impacts. An eco-friendly approach by the manufacturing industries and developing sustainable waste management policies with all-inclusive stakeholders may help build a robust system to face current pandemic waves and future crises.

### 7.1. Effects of Face Mask Waste Materials

The use of face masks is a form of non-pharmaceutical interventions (NPIs) to prevent COVID-19 in conjunction with vaccination and other forms of preventive measures, i.e., social distancing, personal hygiene, and so on. Several scientific reports suggested that healthcare providers, essential workers, service providers, and the general public are exposed to the risk of infection without the proper use of a face mask [120]. In conjunction with mass vaccination, which is a time-consuming and research-intensive task, universal masking is also considered globally to intervene in the rapid transmission of SARS-CoV-2 from human to human and enable a safe transition to achieve herd immunity [72]. However, enabling universal masking possesses an unprecedented impact on our environment, social life, and economy. Face masks produced predominantly from petrochemical-derived non-degradable raw materials cause environmental threats to global daily disposal [120]. Alongside the increasing hazardous waste volume with environmental threats, waste management is an added problem impacting the economy on a national and global scale. Moreover, environmental pollution reduces aesthetic and recreational values impacting social, behavioral, and mental stability [121]. 

#### 7.1.1. Environmental Effects

Face masks are used as the primary PPE to reduce the rapid spread of COVID-19. Thus, with the continuous escalating COVID-19 pandemic, the production and usage of face masks have significantly increased. The extensive use of face masks has two main contributing factors in terms of environmental impact: (i) disposal of waste material and (ii) CO_2_ emission during production. There is a lack of appropriate face mask waste-collecting methods worldwide because of the sudden surge in the use of face masks, and proper disposal methods are being neglected by most people [122]. The vast amount of plastic bodies and particles from disposable face masks end up in streets, landfills, freshwater, and marine water, as people tend to dispose of used face masks recklessly, thus posing a significant threat to the environment. It is estimated from a study conducted in the UK that if each UK citizen disposed of one surgical mask after use daily for a year, that would contribute to an additional 124,000 tons of unrecyclable plastic waste and 57,000 tons of plastic packaging [123]. The PP present in the surgical and N95 masks are 4.5 g and 9 g, respectively, and the USA, Australia, and India generate about 0.6 kt, 0.04 kt, and 2.5 kt PP waste per week, respectively, which substantially impact the waste management and sustainability of the environment [124]. Face mask waste management and handling of potentially hazardous (i.e., contaminated) in this current pandemic have led the world to a more significant challenge. Most face mask waste is plastic-based, chemically stable and difficult to degrade by microorganisms [125]. Moreover, waste incineration and transportation of waste material to designated disposal areas result in energy consumption and greenhouse gas emissions with a serious environmental impact. It was also reported that 10 tons of PPE waste, including face masks, travelled 10 km for the designated disposal area, potentially resulting in a total global warming potential (GWP) impact of 2.76 kg CO_2_-eq [126]. The GWP of municipal solid waste (MSW) and solid medical waste (SMW) is varied from approximately −0.64 to 520 kg CO_2_-eq/ton and −52.1 to 3730 kg CO_2_-eq/ton, respectively, from previous years to recent years in the global pandemic [127]. Moreover, it enters the waterways and reaches fresh water and marine water. This adds the presence of plastics into the aquatic medium.

#### 7.1.2. Social and Behavioral Effects

With the unprecedented rapid spread of COVID-19, it is recommended by the WHO to wear non-medical face masks for healthy people. Since then, several countries have imposed mandatory and voluntary mask policies, which have unknown social and behavioral consequences. These consequences are strongly linked to the efficacy of the measure, stigmatization, and perceived fairness [128]. Using face masks strongly reduces and confuses individuals in emotion recognition, trust attribution, and re-identification, which may significantly impact everyday social interactions and behavioral approaches [129,130]. A study by the National Institute of Standards and Technology (NIST), USA, released a report on the use of mandatory face masks breaking facial recognition algorithms for mass surveillance, which may have national and international security threats [131].

#### 7.1.3. Economic Effects 

Since early 2020, due to nationwide lockdown and global travel restrictions, the tourism industry has experienced significant loss impacting the global economy, and expensive clean-up activities (i.e., sea-shores, lakes, and so on) have an added impact [121]. Besides the environmental impact, waste management and additional workforce may add to the national and global economic impact. MSW and SMW disposal costs varied approximately from USD 90 to 242/ton and USD 12 to 1530/ton, respectively, from previous years to recent years in the pandemic [127]. 

### 7.2. Sustainable Solutions

The current global pandemic has given rise to a spike in biomedical waste generation, including PPE, i.e., surgical and non-surgical face masks, to impact the global environment greatly. A study in the early pandemic has estimated global use of 129 billion face masks monthly, suggesting an additional 30% of waste in the year 2020 compared to the year 2019 [132]. This waste generation imposes physical and chemical pollution due to extensive use of disinfectants, chemical sterilization, and waste incineration. The challenging part of this growing threat is the indiscriminate disposal of face masks and PPE, lack of proper waste management technology in many countries, and limited research on new environmentally friendly biodegradable materials and techniques. The University of Colorado Environmental Center suggested educating oneself on the proper disposable practices and adopting environmentally friendly options, including biodegradable PPE and reusable do-it-yourself (DIY) face masks (e.g., hand-made cloth face masks) [133]. A sustainability check is imperative to determine the economic and environmental viability of a system, i.e., a waste management system that can reduce the risk on investment, thus improving sustainability in the long run. 

#### 7.2.1. Waste Management

COVID-19 has a severe impact on all parts of our society, from the local to the global, and waste management is no exception. Let alone developing countries, COVID-19 has created additional challenges in the waste management sector in developed countries. International organizations and associations have already issued several guidelines for waste management related to infectious diseases and outbreaks. However, only a few guidelines are prepared, particularly in response to the current issue of COVID-19 waste management. These guidelines are being updated and amended in accordance with new scientific findings as the pandemic evolves. However, despite these existing guidelines, it is often difficult for developing counties to follow international standards due to the lack of financial, technical, and social capacities. 

During community outbreaks and global pandemics, waste management is heavily interrupted, leading to improper storage and disposal of wastes. The growing waste build-up and poor waste management may have social, environmental, and health consequences, including the potential transmission of infectious diseases [127]. The Institute for Global Environmental Strategies (IGES) of the United Nations Environment Programme (UNEP) published a waste management report during the COVID-19 pandemic. The report outlined the stages of effective waste management: waste segregation, storage, transportation, treatment, and disposal, with a high emphasis on minimization and recycling of waste material [134]. 

#### 7.2.2. Using Sustainable Materials and Techniques

Technological advancement is crucial for minimizing waste management problems by including corresponding stakeholders from every sector of the waste management systems. Combining these two is the key to developing a sustainable waste management system and policies that may assure resilience in current and future crises [127]. Aside from appropriate disposal and trash management, two major ways to minimize waste materials are reusability and eco-friendliness, i.e., biodegradable PPE or face masks. The mandatory face mask policy and global use of face masks pose serious environmental, social, and economic threats. The current coronavirus disease (COVID-19) has put forward the need for research into biodegradable materials to develop eco-friendly, reusable, and even multifunctional face masks for current and future demands [72].

Biodegradable materials such as biodegradable polymers degrade through natural biological processes into non-toxic gases and carbonaceous soil over time [135]. Thus, the development of complete or partial reusable fibrous face masks (e.g., disposable fibrous filters) based on biodegradable materials can be a viable solution to the environmental problem of waste management. Because of this, there is a significant interest in material research to investigate biodegradable (enzymatically and hydrolytically degradable) materials for medical applications. Similarly, both natural and synthetic biodegradable polymers are being studied to develop biodegradable fibrous material for potential eco-friendly face masks. Synthetic biodegradable polymers, such as polyvinyl alcohol, polyethene oxide, polycaprolactone, and poly-lactic acid, are the main polymers for fiber production using melt-spinning, wet-spinning, and/or electrospinning techniques [72,136]. Recent studies also discussed the possibilities of electrospun nanofibers-based durable and reliable face masks. Electrospun non-woven filters have very small-fiber diameters (less than 100 nm) and can be further processed with disinfection methods and protocols to increase reusability without compromising reliable filterability [136]. Although natural fibers have better biocompatibility and degradability; however, synthetic fibers are easier to process with tailored mechanical properties [135]. Different studies investigated the feasibility of bio-copolymers in terms of better filtration and more functionalities to achieve optimum performance. Both natural and synthetic polymers have been used in combination with clay and inorganic nanoparticles to propose new materials for developing novel filtration media [137,138,139]. Thus, biodegradable polymers are considered potential candidates as raw materials for fabricating future filtration media from a sustainable point of view. Several patented and commercially available biodegradable filtration media for face masks have reportedly been used cellulose, protein, chitosan, gelatin, and PLA in recent years [136]. In addition to biodegradable face masks, several high-performance multifunctional face masks with bioaerosols filtration, antibacterial, and antiviral properties have been reported to be developed in the recent decade. The commercially available ones are mainly non-woven structures incorporating functional materials as required for the face masks to be effective against respiratory diseases [136]. Because most face masks cannot be used repeatedly, it is preferable to use bio-degradable polymeric materials (natural or synthetic) in face masks to ensure their environmental friendliness and viral/bacterial resistance simultaneously. The materials used in bio-degradable face masks are listed in Table 6.

However, De SiO et al. [140] highlighted the possibility of developing environmentally friendly next-generation face masks based on multifunctional membranes, in which the presence of plasmonic nanoparticles and nanoclusters organized in a hierarchical structure enables the destruction of pathogens chemically. Nanomaterials decorated with electrospun nanofibers, such as Cu nanoclusters, are used for their chemically driven intrinsic antiviral and antibacterial activities, whereas Au nanoparticles are shown to be effective photo converters for harnessing synergistically assisted photothermal disinfection. Multilayer electrospun membranes can also dissipate humidity present inside the mask, ensuring a high filtration level and an elevated comfort level, while the photothermal disinfected membrane of the masks has moisture pumping capability.

#### 7.2.3. Education and Social Awareness

The proper disposable practice of a face mask reduces the indiscriminate waste generation and transmission of SARS-CoV-2 and COVID-19. The WHO has published a set of guidelines on ‘How to put on, use, take off, and dispose of a mask?’, which is being updated regularly based on new scientific findings and the evolution of the pandemic. The government and communities demanding people wear masks should introduce explicit education and communication of the benefits of using face masks and mandatory policy to curb community outbreaks during future waves of the global pandemic. The UNEP has suggested a capacity-building and awareness-raising program for COVID-19 waste management for the corresponding stakeholders, such as national and local governments, healthcare facilities, the private sector, and local communities [134].

## 8. Conclusions

This paper reviewed recent advancements in face masks, focusing on their requirements, types, and materials. Additionally, manufacturing techniques, efficacy, challenges, and associated risks are discussed towards their proficient uses and consequences of continuously growing demand for local and global mandates. In addition, the scope of further development in materials studies, new techniques, and advancements towards building a sustainable environment is discussed. The study can be summarized as follows.

Using a face mask in the case of COVID-19 has an obvious medical connotation, although many professional workers use face masks against inhaling dust or harmful substances. Wearing face masks is mandated globally as part of the personal protective and public health measures to curb the rapid transmission of coronavirus disease. Face masks should be used as a comprehensive approach, including social distancing, avoiding close-contact settings and enclosed environment, frequently cleaning hands, and covering sneezes and coughs. Reduction/control of the indiscriminate spread of disease outbreaks in a community requires physical distancing of infected individuals by restricting contacts (i.e., isolation) and protective measures. It is also evident that wearing a face mask curbs the rapid transmissibility of SARS-CoV-2 from human to human per contact by reducing the chances of transmission of infected respiratory particles (i.e., microdroplets) in clinical contexts. Guidelines (published by the WHO) for the use of medical masks (for healthcare providers, anyone who has or may have COVID-19 and anyone who is caring for someone who has or may have COVID-19) and non-medical face masks (for the general population interacting in public gatherings, shopping malls, public transports, and so on) can be implemented to control the outbreak.

Mandatory and universal face masks have several environmental, social, and economic consequences in many phases. Mandatory face mask policy combined with complementary public health measures can reduce community transmission. However, economic experts suggest that mask-wearing mandates add USD 1 trillion to the US GDP.

Medical face masks must meet certain specifications for filtering according to the specified standards and can be further classified with different specifications. In addition to medical face masks, non-medical face masks are also recommended by the WHO.

New biodegradable polymers and/or copolymers (combination of both natural and synthetic polymers) as potential raw materials for the face mask and new techniques are required to be ensured for sustainability. The antibacterial electrospun fibrous mat has great potential in this regard.

Finally, the environmental impact of plastic waste generation from the global spike in the use of face masks currently mostly depends on sustainable waste management, training, and awareness-raising programs for stakeholders and at the social level.

## Figures and Tables

**Table 1:** Comparison of mask standards, ratings, and filtration effectiveness [42].

Mask Type	Standards	Filtration Effectiveness	
**Single-Use** **Face Mask** 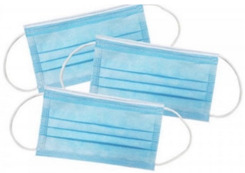	**China**: YY/T0969		
		3.0 Microns: ≥95% 0.1 Microns: **X**	

**Surgical** **Mask** 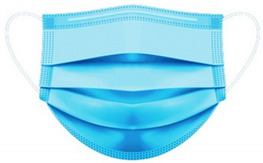	**China**: YY 0469	3.0 Microns: 95%	
		0.1 Microns: 30%	
	**USA**: ASTM F2100	**Level 1**	**Level 2**
		3.0 Microns: ≥95%	3.0 Microns: ≥98%
		0.1 Microns: ≥95%	0.1 Microns: ≥98%
	**Europe**: EN 14683	**Type I**	**Type II**
		3.0 Microns: ≥95%	3.0 Microns: ≥98%
		0.1 Microns: **X**	0.1 Microns: **X**
**Respirator** **Mask** 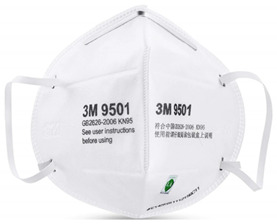	**USA**: NIOSH (42	**N95/KN95**	**N99/KN99**
	CFR 84) **China**: GB2626		
		0.3 Microns: ≥95%	0.3 Microns: ≥99%
		**FFP1**	**FFP2**
	**Europe**: EN 149:2001	0.3 Microns: ≥80%	0.3 Microns: ≥94%

**3.0 Microns:** Bacteria Filtration Efficiency standard (BFE); **0.1 Microns:** Particle Filtration Efficiency standard (PFE); **0.3 Microns:** Used to represent the most-penetrating particle size (MPPS), which is the most difficult size particle to capture; **X**: No requirements.

**Table 2:** Comparison of ASTM F2100-19 standard specification for performance of materials used in medical face masks (USA) and EN 14683:2019 medical face mask requirements and test methods–(EU).

		ASTM F2100-19			EN 14683:2019 Barrier levels		
		Level 1	Level 2	Level 3	Type I	Type II	Type IIR
Barrier testing	BFE (%) ASTM F2101, EN 14683	≥95	≥98		≥95	≥98	
	PFE (%) ASTM F2299	≥95	≥98		Not required		
	Splash resistance, Synthetic blood ASTM F1862, ISO22609	Pass at 80 mmHg	Pass at 120 mmHg	Pass at 160 mmHg	Not required		Pass at ≥ 16.0 kPa (120 mmHg)
Physical testing	Differential pressure EN 14683	5 mmH_2_O/cm^2^	6 mmH_2_O/cm^2^		40 Pa/cm^2^		60 Pa/cm^2^
Safety testing	Flammability 16 CFR Part 1610	Class 1 (≥3.5 seconds)			See European Medical Directive (2007/47/EC, MDD 93/42/EEC)		
	Microbial cleanliness ISO 11737-1	Not required			≤30 cfu/g		
	Biocompatibility ISO 10993	510 k Guidance recommends testing to ISO 10993			Complete an evaluation according to ISO 10993		
Sampling ANSI/ASQC Z1.4 ISO 2859-1		▪ AQL 4% for BFE, PFE, delta P ▪ 32 masks for synthetic blood (Pass ≥29 passing, Fail ≤28 passing) ▪ 14 masks for flammability			▪ Minimum of 5 masks up to an AQL of 4% for BFE, delta P and microbial cleanliness ▪ 32 masks for synthetic blood splash resistance (Pass ≥ 29 passing, Fail ≤ 28 passing)		

**Table 3:** Commonly used mask materials and their products and properties [45].

Polymers		Products	Properties
Polyolefin	Polypropylene (PP)	Nonwoven melt blown and spunbond fibers	Low cost, lightest weight among all synthetic fabrics due to its low density and specific gravity, ability to filter dry particulates, high chemical (alkali and acid) resistance, ease of processing, recyclability, modifiable inherent hydrophobicity, good mechanical strength, abrasion resistance, and micropore distribution uniformity make PP a promising option for manufacturing face masks. PP has higher mechanical strength and is less expensive than PE.
	Polyethylene (PE)	Meltblown nonwoven fibers	PE with different densities, including high-density PE, low-density PE, and linear low-density PE, can be made. Good chemical resistance, lightweight, and hydrophobic. PE is easier to extrude than PP due to the high shear sensitivity and higher melting temperature of PP.
Polyesters	Polyethylene terephthalate (PET)	Spunbond nonwoven fibers	Higher tensile modulus, strength, and heat stability, but less cost-effective than PP and more difficult to recycle.
Polyamide	Nylon 6 and 6–6	Spunbond nonwoven fabrics	Fiber lightness and high melting temperature (260 °C), but unsuitable for face masks due to water absorption.
Cellulose Acetate (CA)		Electrospun nanofibrous membranes	High filtration efficacy, low thickness, hydrophobic, low production cost, biodegradable, high water stability, but soluble in organic solvents.
Poly- (vinyl alcohol) (PVA)		Nanofibrous membranes	Lightweight, biodegradable, cost-effective, washable, and reusable.
Polylactic Acid (PLA)		Nanofibrous membranes	Biodegradable, cost-effective, favorable mechanical properties, and filtration efficiency of 99.99%.
Polytetrafluoroethylene (PTFE)		Air filter membranes	Lightweight, hydrophobic, great chemical stability, high surface fracture toughness, and high heat resistance. Because of its strong C-C and C-F bonds, PTEF membrane is extensively utilized as an air filter membrane with high filtration and fine particle rejection rate of greater than 99.99%.
Polyacrylonitrile (PAN)		Waterproof membranes	High cost, significant variations in fiber diameters and mat morphologies, chemical and thermal stabilizations.

**Table 4:** Filtration efficacy of different mask fabrics [76].

Material	Source	Structure	Basis Weight (gm^−2^)	Bulk Density (Basis Weight/Thickness) (gm^−2^µm^−1^)	Initial Filtration Efficiency (%)	Initial Pressure Drop (Pa)	Filter Quality Factor, Q (kPa^−1^)
Personal Protection Materials							
PP 1	Particulate FFR	Meltblown (nonwoven)	25	0.17	95.94 ± 2	9.0 ± 2.0	162.7 ± 21.3
PP 2	Medical face mask	Meltblown (nonwoven)	26	0.21	33.06 ± 0.95	34.3 ± 0.5	5 ± 0.1
PP 3	Medical face mask	Meltblown (nonwoven)	20	0.20	18.81 ± 0.5	16.3 ± 0.5	5.5 ± 0.1
Household Materials							
PP 4	Interfacing material, purchased as-is	Spunbond (Nonwoven)	30	0.26	6.15 ± 2.18	1.6 ± 0.5	16.9 ± 3.4
Cotton 1	Clothing (T-shirt)	Woven	116	0.57	5.04 ± 0.64	4.5 ± 2.1	5.4 ± 1.9
Cotton 2	Clothing (T-shirt)	Knit	157	0.37	21.62 ± 1.84	14.5 ± 2.1	7.4 ± 1.7
Cotton 3	Clothing (Sweater)	Knit	360	0.45	25.88 ± 1.41	17 ± 0.0	7.6 ± 0.4
Polyester	Clothing (Toddler wrap)	Knit	200	0.38	17.50 ± 5.10	12.3 ± 0.5	6.8 ± 2.4
Silk	Napkin	Woven	84	0.54	4.77 ± 1.47	7.3 ± 1.5	2.8 ± 0.4
Nylon	Clothing (Exercise pants)	Woven	164	0.70	23.33 ± 1.18	244 ± 5.5	0.4 ± 0.0
Cellulose	Paper towel	Bonded	42.9	0.33	10.41 ± 0.28	11 ± 0.0	4.3 ± 2.8
Cellulose	Tissue paper	Bonded	32.8	0.39	20.2 ± 0.32	19 ± 1	5.1 ± 3.2
Cellulose	Copy paper	Bonded	72.8	0.76	99.85 ± 0.02	1883.6 ± 39.3	1.5 ± 0.2

**Table 5:** General characteristics of different types of masks [119].

Mask Type	Surgical	N-95	Cloth/Fabric
	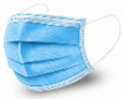	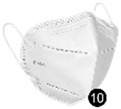	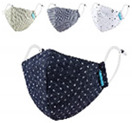
Fibers	Polypropylene Rayon Polyethylene Polyacrylonitrile Polyester Polyolefin Thermoplastic polymer	Cellulosic natural fiber Polypropylene Polytetrafluoroethylene Polyvinylidene fluoride Cellulose acetate	Densely woven fabric and knitted jersey fabric of natural cotton Silk fibers Pillowcase Kitchen towels Tea cloths
Characteristics	SMS non-woven, loose fitting, health worker wears readily, 3-layer, total 60–70 GSM	Facile tight-fitting, smart valve, efficient filtration of airborne particles (more than 95%), very high GSM	Used by the general population, any layer and fabric of different GSM can be assembled quickly, 3–4 layers, moderate GSM (250–450)
Benefits	Good filtration ability (more than 80%), cheap, single time use	For health personnel, efficient protection (MERS, SARS, Avian Flu, Ebola Virus, and PM2.5), good protection against COVID-19	Easily made, homemade cloth reuse, washable, good fit
Shortcomings	Air leakage, a lot of copy products in the market	Uncomfortable to use, high cost	Not very efficient against COVID-19, insufficient protection to aerosols
Suggestions	▪ Manufacturers and vendors should strive to produce a quality standard mask to prevent viruses from entering the body through the nose. ▪ It is necessary to educate the public about mask guidelines and disinfection methods. ▪ Used masks should be kept away from children and other vulnerable people.		

**Table 6:** Bio-based materials for the surgical face mask [72].

Bio-Based Media	Structure and Materials	Applications
Protein	Keratin/polyamide 6 nanofiber	Water and air filtration
	Electrospun sericin nanofibrous mats	Air filtration mask
	Silk nanofibers	Air filtration mask
	Gluten nanofiber	Face mask
	Soy protein isolate/PVA hybrid nanofiber	Air filtration mask
Cellulose	Nanomembrane lyocell fibers	Surgical face mask
	Cellulose non-woven layers	Surgical face mask
	Cellulose acetate nanofibers	Air filtration
	3-ply cotton-PLA-cotton layered	Face mask
	Fungal hyphae and cellulose fibers (wood and hemp)	Alternative to synthetic melt and spun-blown materials for PPE
	Banana stem fiber	Face mask
	Non-woven cellulosic fiber	Face mask
Chitosan	Nanofibrous chitosan non-woven	Water and air filtration
Poly lactic acid	Poly lactic acid fibrous membranes	Air filtration
	3D printed and electrospun polylactic acid	Face mask filter
Gelatin	Gelatin/β–cyclodextrin composite nanofiber	Respiratory filter

## Data Availability

Not applicable.

## References

[1] Wei Z.-Y., Qiao R., Chen J., Huang J., Wu H., Wang W.-J., Yu H., Xu J., Wang C., Gu C.-H. (2021). The influence of pre-existing hypertension on coronavirus disease 2019 patients. Epidemiol. Infect..

[2] (2019). Coronavirus Disease (COVID-19) Pandemic. https://www.who.int/emergencies/diseases/novel-coronavirus-2019.

[3] WHO (2020). Coronavirus (COVID-19) Dashboard. https://covid19.who.int/.

[4] Abera A., Belay H., Zewude A., Gidey B., Nega D., Dufera B., Abebe A., Endriyas T., Getachew B., Birhanu H. (2020). Establishment of COVID-19 testing laboratory in resource-limited settings: Challenges and prospects reported from Ethiopia. Glob. Health Action.

[5] Rodríguez-Rey R., Garrido-Hernansaiz H., Collado S. (2020). Psychological impact and associated factors during the initial stage of the coronavirus (COVID-19) pandemic among the general population in Spain. Front. Psychol..

[6] Gupta M.D., Girish M., Yadav G., Shankar A., Yadav R. (2020). Coronavirus disease 2019 and the cardiovascular system: Impacts and implications. Indian Heart J..

[7] Giannis D., Geropoulos G., Matenoglou E., Moris D. (2021). Impact of coronavirus disease 2019 on healthcare workers: Beyond the risk of exposure. Postgrad. Med. J..

[8] Rahman T., Khandakar A., Hoque M.E., Ibtehaz N., Kashem S.B., Masud R., Shampa L., Hasan M.M., Islam M.T., Al-Madeed S. (2021). Development and Validation of an Early Scoring System for Prediction of Disease Severity in COVID-19 using Complete Blood Count Parameters. IEEE Access.

[9] Gorain B., Choudhury H., Molugulu N., Athawale R.B., Kesharwani P. (2020). Fighting strategies against the novel coronavirus pandemic: Impact on global economy. Front. Public Health.

[10] Bhat B.A., Khan S., Manzoor S., Niyaz A., Tak H., Anees S., Gull S., Ahmad I. (2020). A study on impact of COVID-19 lockdown on psychological health, economy and social life of people in Kashmir. Int. J. Sci. Healthc. Res..

[11] Sohrabi C., Alsafi Z., O’neill N., Khan M., Kerwan A., Al-Jabir A., Iosifidis C., Agha R. (2020). World Health Organization declares global emergency: A review of the 2019 novel coronavirus (COVID-19). Int. J. Surg..

[12] Shi L., Lu Z.-A., Que J.-Y., Huang X.-L., Liu L., Ran M.-S., Gong Y.-M., Yuan K., Yan W., Sun Y.-K. (2020). Prevalence of and risk factors associated with mental health symptoms among the general population in China during the coronavirus disease 2019 pandemic. JAMA Netw. Open.

[13] Sabino-Silva R., Jardim A.C.G., Siqueira W.L. (2020). Coronavirus COVID-19 impacts to dentistry and potential salivary diagnosis. Clin. Oral Investig..

[14] Hiscott J., Alexandridi M., Muscolini M., Tassone E., Palermo E., Soultsioti M., Zevini A. (2020). The global impact of the coronavirus pandemic. Cytokine Growth Factor Rev..

[15] Ares G., Bove I., Vidal L., Brunet G., Fuletti D., Arroyo Á., Blanc M.V. (2021). The experience of social distancing for families with children and adolescents during the coronavirus (COVID-19) pandemic in Uruguay: Difficulties and opportunities. Child. Youth Serv. Rev..

[16] Ganesan B., Al-Jumaily A., Fong K.N., Prasad P., Meena S.K., Tong R.K.-Y. (2021). Impact of Coronavirus Disease 2019 (COVID-19) outbreak quarantine, isolation, and lockdown policies on mental health and suicide. Front. Psychiatry.

[17] Qiu Y., Chen X., Shi W. (2020). Impacts of social and economic factors on the transmission of coronavirus disease 2019 (COVID-19) in China. J. Popul. Econ..

[18] Milman E., Lee S.A., Neimeyer R.A. (2020). Social isolation and the mitigation of coronavirus anxiety: The mediating role of meaning. Death Stud..

[19] Helm D. (2020). The environmental impacts of the coronavirus. Environ. Resour. Econ..

[20] Sarkodie S.A., Owusu P.A. (2021). Global assessment of environment, health and economic impact of the novel coronavirus (COVID-19). Environ. Dev. Sustain..

[21] Isaifan R. (2020). The dramatic impact of Coronavirus outbreak on air quality: Has it saved as much as it has killed so far?. Glob. J. Environ. Sci. Manag..

[22] Rupani P.F., Nilashi M., Abumalloh R., Asadi S., Samad S., Wang S. (2020). Coronavirus pandemic (COVID-19) and its natural environmental impacts. Int. J. Environ. Sci. Technol..

[23] Sukharev O.S. (2020). Economic crisis as a consequence COVID-19 virus attack: Risk and damage assessment. Quant. Financ. Econ..

[24] Grignoli N., Petrocchi S., Bernardi S., Massari I., Traber R., Malacrida R., Gabutti L. (2020). Influence of empathy disposition and risk perception on the psychological impact of lockdown during the coronavirus disease pandemic outbreak. Front. Public Health.

[25] Howard J., Huang A., Li Z., Tufekci Z., Zdimal V., van der Westhuizen H.-M., von Delft A., Price A., Fridman L., Tang L.-H. (2021). An evidence review of face masks against COVID-19. Proc. Natl. Acad. Sci. USA.

[26] WHO Coronavirus Disease (COVID-19) Advice for the Public: When and How to Use Masks. https://www.who.int/emergencies/diseases/novel-coronavirus-2019/advice-for-public/when-and-how-to-use-masks.

[27] Wu L.-T. (1926). A treatise on pneumonic plague. A Treatise on Pneumonic Plague.

[28] Goh L., Ho T., Phua K. (1987). Wisdom and western science: The work of Dr Wu Lien-Teh. Asia Pac. J. Public Health.

[29] Wang Y., Tian H., Zhang L., Zhang M., Guo D., Wu W., Zhang X., Kan G.L., Jia L., Huo D. (2020). Reduction of secondary transmission of SARS-CoV-2 in households by face mask use, disinfection and social distancing: A cohort study in Beijing, China. BMJ Glob. Health.

[30] Usher Institute, The University of Edinburgh (2020). Does the Use of Face Masks in the General Population Make a Difference to Spread of Infection?. https://www.ed.ac.uk/files/atoms/files/uncover_003-03_summary_-_facemasks_community_anon.pdf.

[31] Gupta M., Gupta K., Gupta S. (2020). The use of face masks by the general population to prevent transmission of COVID-19 infection: A systematic review. medRxiv.

[32] Chu D.K., Akl E.A., Duda S., Solo K., Yaacoub S., Schünemann H.J., El-harakeh A., Bognanni A., Lotfi T., Loeb M. (2020). Physical distancing, face masks, and eye protection to prevent person-to-person transmission of SARS-CoV-2 and COVID-19: A systematic review and meta-analysis. Lancet.

[33] Wu J., Xu F., Zhou W., Feikin D.R., Lin C.-Y., He X., Zhu Z., Liang W., Chin D.P., Schuchat A. (2004). Risk factors for SARS among persons without known contact with SARS patients, Beijing, China. Emerg. Infect. Dis..

[34] Jefferson T., Del Mar C.B., Dooley L., Ferroni E., Al-Ansary L.A., Bawazeer G.A., Van Driel M.L., Jones M.A., Thorning S., Beller E.M. (2020). Physical interventions to interrupt or reduce the spread of respiratory viruses. Cochrane Database Syst. Rev..

[35] MacIntyre C.R., Chughtai A.A. (2020). A rapid systematic review of the efficacy of face masks and respirators against coronaviruses and other respiratory transmissible viruses for the community, healthcare workers and sick patients. Int. J. Nurs. Stud..

[36] MacIntyre C.R., Dwyer D., Seale H., Fasher M., Booy R., Cheung P., Ovdin N., Browne G. (2008). The first randomized, controlled clinical trial of mask use in households to prevent respiratory virus transmission. Int. J. Infect. Dis..

[37] Suess T., Remschmidt C., Schink S.B., Schweiger B., Nitsche A., Schroeder K., Doellinger J., Milde J., Haas W., Koehler I. (2012). The role of facemasks and hand hygiene in the prevention of influenza transmission in households: Results from a cluster randomised trial; Berlin, Germany, 2009–2011. BMC Infect. Dis..

[38] Cowling B.J., Chan K.-H., Fang V.J., Cheng C.K., Fung R.O., Wai W., Sin J., Seto W.H., Yung R., Chu D.W. (2009). Face masks and hand hygiene to prevent influenza transmission in households: A cluster randomized trial. Ann. Intern. Med..

[39] Aiello A.E., Murray G.F., Perez V., Coulborn R.M., Davis B.M., Uddin M., Shay D.K., Waterman S.H., Monto A.S. (2010). Mask use, hand hygiene, and seasonal influenza-like illness among young adults: A randomized intervention trial. J. Infect. Dis..

[40] Aiello A.E., Perez V., Coulborn R.M., Davis B.M., Uddin M., Monto A.S. (2012). Face masks, hand hygiene, and influenza among young adults: A randomized intervention trial. PLoS ONE.

[41] Higgins J.P., Thomas J., Chandler J., Cumpston M., Li T., Page M.J., Welch V.A. (2019). Cochrane Handbook for Systematic Reviews of Interventions.

[42] Robertson P. (2021). Comparison of Mask Standards, Ratings, and Filtration Effectiveness. https://smartairfilters.com/en/blog/comparison-mask-standards-rating-effectiveness/.

[43] Ghosh S. (2014). Composite nonwovens in medical applications. Compos. Non-Woven Mater..

[44] Garcia R.A., Stevanovic T., Berthier J., Njamen G., Tolnai B., Achim A. (2021). Cellulose, Nanocellulose, and Antimicrobial Materials for the Manufacture of Disposable Face Masks: A Review. BioResources.

[45] Ogbuoji E.A., Zaky A.M., Escobar I.C. (2021). Advanced Research and Development of Face Masks and Respirators Pre and Post the Coronavirus Disease 2019 (COVID-19) Pandemic: A Critical Review. Polymers.

[46] Konda A., Prakash A., Moss G.A., Schmoldt M., Grant G.D., Guha S. (2020). Aerosol Filtration Efficiency of Common Fabrics Used in Respiratory Cloth Masks. ACS Nano.

[47] (2021). Occupational Exposure to COVID-19; Emergency Temporary Standard. https://www.federalregister.gov/documents/2021/06/21/2021-12428/occupational-exposure-to-covid-19-emergency-temporary-standard.

[48] 3M Company (2021). Comparison of P2, FFP2, KN95, and N95 and Other Filtering Facepiece Respirator Classes.

[49] (2021). NIOSH-Approved Particulate Filtering Facepiece Respirators. https://www.cdc.gov/niosh/npptl/topics/respirators/disp_part/default.html.

[50] Ottawa Public Health (2022). Ways to Have Better Fit and Extra Protection with Cloth and Disposable Masks.

[51] Matuschek C., Moll F., Fangerau H., Fischer J.C., Zänker K., van Griensven M., Schneider M., Kindgen-Milles D., Knoefel W.T., Lichtenberg A. (2020). Face masks: Benefits and risks during the COVID-19 crisis. Eur. J. Med. Res..

[52] Armentano I., Barbanera M., Carota E., Crognale S., Marconi M., Rossi S., Rubino G., Scungio M., Taborri J., Calabro G. (2021). Polymer materials for respiratory protection: Processing, end use, and testing methods. ACS Appl. Polym. Mater..

[53] Wibisono Y., Fadila C.R., Saiful S., Bilad M.R. (2020). Facile approaches of polymeric face masks reuse and reinforcements for micro-aerosol droplets and viruses filtration: A review. Polymers.

[54] Li J., Wang W., Jiang R., Guo C. (2021). Antiviral Electrospun Polymer Composites: Recent Advances and Opportunities for Tackling COVID-19. Front. Mater..

[55] Rahman M.Z. (2022). Influence of Fiber Treatment on the Damping Performance of Plant Fiber Composites. Vibration and Damping Behavior of Biocomposites.

[56] Rahman M.Z., Mace B.R., Jayaraman K. Vibration damping of natural fibre-reinforced composite materials. Proceedings of the 17th European Conference on Composite Material.

[57] Selvaranjan K., Navaratnam S., Rajeev P., Ravintherakumaran N. (2021). Environmental challenges induced by extensive use of face masks during COVID-19: A review and potential solutions. Environ. Chall..

[58] Rahman M.Z. (2021). Mechanical and damping performances of flax fibre composites–A review. Compos. Part C Open Access.

[59] Rahman M.Z., Jayaraman K., Mace B.R. (2017). Vibration damping of flax fibre-reinforced polypropylene composites. Fibers Polym..

[60] Seidi F., Deng C., Zhong Y., Liu Y., Huang Y., Li C., Xiao H. (2021). Functionalized Masks: Powerful Materials against COVID-19 and Future Pandemics. Small.

[61] Rahman M.Z., Jayaraman K., Mace B.R. (2018). Impact energy absorption of flax fiber-reinforced polypropylene composites. Polym. Compos..

[62] Rahman M.Z., Jayaraman K., Mace B.R. (2018). Influence of damping on the bending and twisting modes of flax fibre-reinforced polypropylene composite. Fibers Polym..

[63] Jung S., Lee S., Dou X., Kwon E.E. (2021). Valorization of disposable COVID-19 mask through the thermo-chemical process. Chem. Eng. J..

[64] Rahman M.Z. (2013). Fabrication, Morphologies and Properties of Single Polymer Composites Based on LLDPE, PP, and CNT Loaded PBT.

[65] Rahman M.Z. (2017). Static and Dynamic Characterisation of Flax Fibre-Reinforced Polypropylene Composites. Ph.D. Thesis.

[66] Amini G., Karimi M., Zokaee Ashtiani F. (2020). Hybrid electrospun membrane based on poly (vinylidene fluoride)/poly (acrylic acid)–poly (vinyl alcohol) hydrogel for waterproof and breathable applications. J. Ind. Text..

[67] O’Dowd K., Nair K.M., Forouzandeh P., Mathew S., Grant J., Moran R., Bartlett J., Bird J., Pillai S.C. (2020). Face masks and respirators in the fight against the COVID-19 pandemic: A review of current materials, advances and future perspectives. Materials.

[68] Adanur S., Jayswal A. (2020). Filtration mechanisms and manufacturing methods of face masks: An overview. J. Ind. Text..

[69] Kulmala I., Heinonen K., Salo S. (2021). Improving Filtration Efficacy of Medical Face Masks. Aerosol Air Qual. Res..

[70] Shimasaki N., Okaue A., Morimoto M., Uchida Y., Koshiba T., Tsunoda K., Arakawa S., Shinohara K. (2020). A multifaceted evaluation on the penetration resistance of protective clothing fabrics against viral liquid drops without pressure. Biocontrol Sci..

[71] Chua M.H., Cheng W., Goh S.S., Kong J., Li B., Lim J.Y., Mao L., Wang S., Xue K., Yang L. (2020). Face masks in the new COVID-19 normal: Materials, testing, and perspectives. Research.

[72] Babaahmadi V., Amid H., Naeimirad M., Ramakrishna S. (2021). Biodegradable and multifunctional surgical face masks: A brief review on demands during COVID-19 pandemic, recent developments, and future perspectives. Sci. Total Environ..

[73] Mukhopadhyay A. (2014). Composite nonwovens in filters: Applications. Composite Non-Woven Materials.

[74] Davison A.M. (2012). Pathogen inactivation and filtration efficacy of a new anti-microbial and anti-viral surgical facemask and N95 against dentistry-associated microorganisms. Int. Dent. Australas. Ed..

[75] Chellamani K., Veerasubramanian D., Balaji R.V. (2013). Surgical face masks: Manufacturing methods and classification. J. Acad. Ind. Res..

[76] Zhao M., Liao L., Xiao W., Yu X., Wang H., Wang Q., Lin Y.L., Kilinc-Balci F.S., Price A., Chu L. (2020). Household materials selection for homemade cloth face coverings and their filtration efficiency enhancement with triboelectric charging. Nano Lett..

[77] Essa W.K., Yasin S.A., Saeed I.A., Ali G.A. (2021). Nanofiber-Based Face Masks and Respirators as COVID-19 Protection: A Review. Membranes.

[78] Epps H.H., Leonas K.K. (2000). Pore size and air permeability of four nonwoven fabrics. Int. Nonwovens J..

[79] Verma D. (2021). Effectiveness of Masks: Fast Answers with Automated SEM Analysis.

[80] Nuge T., Tshai K.Y., Lim S.S., Nordin N., Hoque M.E. (2020). Characterization and optimization of the mechanical properties of electrospun gelatin nanofibrous scaffolds. World J. Eng..

[81] Majumder S., Sharif A., Hoque M.E., Faris M.A., Sapuan S.M. (2020). Electrospun cellulose acetate nanofiber: Characterization and applications. Advanced Processing, Properties, and Applications of Starch and Other Bio-Based Polymers.

[82] Pradeep S.A., Kumar G.P., Phani A.R., Prasad R.G.S.V., Hoque M.E., Raghavendra H.L. (2015). Fabrication, characterization and in vitro osteogenic potential of polyvinyl pyrrolidone-titania (PVP-TiO) nanofibers. Anal. Chem. Lett..

[83] Hoque M.E., Peiris A.M., Rahman S.M.A., Wahab M.A. (2018). New Generation Antibacterial Nanofibrous Membrane For Potential Water Filtration. Curr. Anal. Chem..

[84] Alayande A.B., Kang Y., Jang J., Jee H., Lee Y.-G., Kim I.S., Yang E. (2021). Antiviral Nanomaterials for Designing Mixed Matrix Membranes. Membranes.

[85] Palmieri L., Vanacore N., Donfrancesco C., Lo Noce C., Canevelli M., Punzo O., Raparelli V., Pezzotti P., Riccardo F., Bella A. (2020). Clinical characteristics of hospitalized individuals dying with COVID-19 by age group in Italy. J. Gerontol. Ser. A..

[86] Stan A., Steiner S., Majeed S., Weber S.S., Gosh S., Semren T.Ž., Guy P.A., Lebrun S., Steinhauser J., Tardy Y. (2021). Aerosol filtration testing of fabrics for development of reusable face masks. Aerosol Air Qual. Research.

[87] Tsai P.P., Schreuder-Gibson H., Gibson P. (2002). Different electrostatic methods for making electret filters. J. Electrost..

[88] Chowdhury M.A., Shuvho M.B., Shahid M.A., Haque A.M., Kashem M.A., Lam S.S., Ong H.C., Uddin M.A., Mofijur M. (2021). Prospect of biobased antiviral face mask to limit the coronavirus outbreak. Environ. Res..

[89] Jain M., Kim S.T., Xu C., Li H., Rose G. (2020). Efficacy and use of cloth masks: A scoping review. Cureus.

[90] Darby S., Chulliyallipalil K., Przyjalgowski M., McGowan P., Jeffers S., Giltinan A., Lewis L., Smith N., Sleator R.D. (2021). COVID-19: Mask efficacy is dependent on both fabric and fit. Future Microbiol..

[91] Gierthmuehlen M., Kuhlenkoetter B., Parpaley Y., Gierthmuehlen S., Köhler D., Dellweg D. (2020). Evaluation and discussion of handmade face-masks and commercial diving-equipment as personal protection in pandemic scenarios. PLoS ONE.

[92] Formentini G., Rodríguez N.B., Favi C., Marconi M. (2021). Challenging the engineering design process for the development of facial masks in the constraint of the COVID-19 pandemic. Procedia Cirp..

[93] Lindsley W.G., Blachere F.M., Law B.F., Beezhold D.H., Noti J.D. (2021). Efficacy of face masks, neck gaiters and face shields for reducing the expulsion of simulated cough-generated aerosols. Aerosol Sci. Technol..

[94] Mboowa G., Semugenze D., Nakabuye H., Bulafu D., Aruhomukama D. (2021). Efficacy of Face Masks Used in Uganda: A Laboratory-Based Inquiry during the COVID-19 Pandemic. Am. J. Trop. Med. Hyg..

[95] Sharma S., Pinto R., Saha A., Chaudhuri S., Basu S. (2021). On secondary atomization and blockage of surrogate cough droplets in single-and multilayer face masks. Sci. Adv..

[96] Teesing G.R., van Straten B., de Man P., Horeman-Franse T. (2020). Is there an adequate alternative to commercially manufactured face masks? A comparison of various materials and forms. J. Hosp. Infect..

[97] Wilson A.M., Abney S.E., King M.F., Weir M.H., López-García M., Sexton J.D., Dancer S.J., Proctor J., Noakes C.J., Reynolds K.A. (2020). COVID-19 and use of non-traditional masks: How do various materials compare in reducing the risk of infection for mask wearers?. J. Hosp. Infect..

[98] Fischer E.P., Fischer M.C., Grass D., Henrion I., Warren W.S., Westman E. (2020). Low-cost measurement of face mask efficacy for filtering expelled droplets during speech. Sci. Adv..

[99] Aydin O., Emon B., Cheng S., Hong L., Chamorro L.P., Saif M.T.A. (2020). Performance of fabrics for home-made masks against the spread of COVID-19 through droplets: A quantitative mechanistic study. Extrem. Mech. Lett..

[100] Serfozo N., Ondrácek J., Zíková N., Lazaridis M., Ždímal V. (2017). Size-resolved penetration of filtering materials from CE-marked filtering facepiece respirators. Aerosol Air Qual. Res..

[101] Ippolito M., Vitale F., Accurso G., Iozzo P., Gregoretti C., Giarratano A., Cortegiani A. (2020). Medical masks and Respirators for the Protection of Healthcare Workers from SARS-CoV-2 and other viruses. Pulmonology.

[102] Scully J.R., Hutchison M., Santucci R.J. (2021). The *COVID-19* Pandemic, Part 2: Understanding the efficacy of oxidized copper compounds in suppressing infectious aerosol-based virus transmission. Corrosion.

[103] Bhimaraju H., Jain R., Nag N. (2020). Low-Cost Enhancement of Facial Mask Filtration to Prevent Transmission of COVID-19. Biom. Biostat. Int. J..

[104] Lima M.M.D.S., Cavalcante F.M.L., Macêdo T.S., Galindo-Neto N.M., Caetano J.Á., Barros L.M. (2020). Cloth face masks to prevent *COVID-19* and other respiratory infections. Rev. Lat. Am. De Enferm..

[105] Grover C. (2020). Efficacy of face masks depends on spatial relation between host and recipient and who is being protected. BMJ.

[106] Ronen A., Rotter H., Elisha S., Sevilia S., Parizer B., Hafif N., Manor A. (2021). Investigation of the protection efficacy of face shields against aerosol cough droplets. J. Occup. Environ. Hyg..

[107] Jefferson T., Foxlee R., Del Mar C., Dooley L., Ferroni E., Hewak B., Prabhala A., Nair S., Rivetti A. (2008). Physical interventions to interrupt or reduce the spread of respiratory viruses: Systematic review. BMJ.

[108] Zhou S.S., Lukula S., Chiossone C., Nims R.W., Suchmann D.B., Ijaz M.K. (2018). Assessment of a respiratory face mask for capturing air pollutants and pathogens including human influenza and rhinoviruses. J. Thorac. Dis..

[109] Verbeek J.H., Rajamaki B., Ijaz S., Sauni R., Toomey E., Blackwood B., Tikka C., Ruotsalainen J.H., Balci F.S.K. (2020). Personal protective equipment for preventing highly infectious diseases due to exposure to contaminated body fluids in healthcare staff. Cochrane Database Syst. Rev..

[110] Tirupathi R., Bharathidasan K., Palabindala V., Salim S.A., Al-Tawfiq J.A. (2020). Comprehensive review of mask utility and challenges during the *COVID-19* pandemic. Infez Med..

[111] Tucho G.T., Kumsa D.M. (2021). Universal Use of Face Masks and Related Challenges During COVID-19 in Developing Countries. Risk Manag. Healthc. Policy.

[112] De Man P., van Straten B., van den Dobbelsteen J., Van Der Eijk A., Horeman T., Koeleman H. (2020). Sterilization of disposable face masks by means of standardized dry and steam sterilization processes; an alternative in the fight against mask shortages due to COVID-19. J. Hosp. Infect..

[113] European Centre for Disease Prevention and Control (2020). Cloth Masks and Mask Sterilisation as Options in Case of Shortage of Surgical Masks and Respirators.

[114] Lowe J.J., Paladino K.D., Farke J.D., Boulter K., Cawcutt K., Emodi M., Gibbs S., Hankins R., Hinkle L., Micheels T. (2020). N95 Filtering Facepiece Respirator Ultraviolet Germicidal Irradiation (Uvgi) Process for Decontamination and Reuse.

[115] Ma Q.X., Shan H., Zhang C.M., Zhang H.L., Li G.M., Yang R.M., Chen J.M. (2020). Decontamination of face masks with steam for mask reuse in fighting the pandemic COVID-19: Experimental supports. J. Med. Virol..

[116] Schwartz A., Stiegel M., Greeson N., Vogel A., Thomann W., Brown M., Sempowski G.D., Alderman T.S., Condreay J.P., Burch J. (2020). Decontamination and reuse of N95 respirators with hydrogen peroxide vapor to address worldwide personal protective equipment shortages during the SARS-CoV-2 (*COVID-19*) pandemic. Appl. Biosaf..

[117] Wehlage D., Blattner H., Sabantina L., Böttjer R., Grothe T., Rattenholl A., Gudermann F., Lütkemeyer D., Ehrmann A. (2019). Sterilization of PAN/gelatin nanofibrous mats for cell growth. Tekstilec.

[118] Mackenzie D. (2020). Reuse of N95 Masks. Engineering.

[119] Coffey C., D’Alessandro M.M., Cichowicz J.K. Respiratory Protection During Outbreaks: Respirators versus Surgical Mask. https://blogs.cdc.gov/niosh-science-blog/2020/04/09/masks-v-respirators/.

[120] Das O., Neisiany R.E., Capezza A.J., Hedenqvist M.S., Försth M., Xu Q., Jiang L., Ji D., Ramakrishna S. (2020). The need for fully bio-based facemasks to counter coronavirus outbreaks: A perspective. Sci. Total Environ..

[121] Zhang D., Liu X., Huang W., Li J., Wang C., Zhang D., Zhang C. (2020). Microplastic pollution in deep-sea sediments and organisms of the Western Pacific Ocean. Environ. Pollut..

[122] Sangkham S. (2020). Face mask and medical waste disposal during the novel COVID-19 pandemic in Asia. Case Stud. Chem. Environ. Eng..

[123] Allison A.L., Ambrose-Dempster E., Aparsi T.D., Bawn M., Casas Arredondo M., Chau C., Chandler K., Dobrijevic D., Hailes H., Lettieri P. (2020). The Environmental Dangers of Employing Single-Use Face Masks as Part of a COVID-19 Exit Strategy.

[124] Abbasi S.A., Khalil A.B., Arslan M. (2020). Extensive use of face masks during COVID-19 pandemic:(micro-) plastic pollution and potential health concerns in the Arabian Peninsula. Saudi J. Biol. Sci..

[125] Webb H.K., Arnott J., Crawford R.J., Ivanova E.P. (2013). Plastic degradation and its environmental implications with special reference to poly (ethylene terephthalate). Polymers.

[126] Kumar H., Azad A., Gupta A., Sharma J., Bherwani H., Labhsetwar N.K., Kumar R. (2021). COVID-19 Creating another problem? Sustainable solution for PPE disposal through LCA approach. Environ. Dev. Sustain..

[127] Roy P., Mohanty A.K., Wagner A., Sharif S., Khalil H., Misra M. (2021). Impacts of COVID-19 Outbreak on the Municipal Solid Waste Management: Now and beyond the Pandemic. ACS Environ. Au..

[128] Betsch C., Korn L., Sprengholz P., Felgendreff L., Eitze S., Schmid P., Böhm R. (2020). Social and behavioral consequences of mask policies during the COVID-19 pandemic. Proc. Natl. Acad. Sci. USA.

[129] Carbon C.-C. (2020). Wearing face masks strongly confuses counterparts in reading emotions. Front. Psychol..

[130] Marini M., Ansani A., Paglieri F., Caruana F., Viola M. (2021). The impact of facemasks on emotion recognition, trust attribution and re-identification. Sci. Rep..

[131] NIST (2020). NIST Launches Studies into Masks’ Effect on Face Recognition Software.

[132] Prata J.C., Silva A.L., Walker T.R., Duarte A.C., Rocha-Santos T. (2020). COVID-19 pandemic repercussions on the use and management of plastics. Environ. Sci. Technol..

[133] Conzachi K. (2020). What You Can Do to Reduce the Environmental Impacts of COVID-19. https://www.colorado.edu/ecenter/2020/11/13/what-you-can-do-reduce-environmental-impacts-COVID-1.

[134] Tsukiji M., Gamaralalage P.J.D., Pratomo I.S.Y., Onogawa K., Alverson K., Honda S., Ternald D., Dilley M., Fujioka J., Condrorini D. (2020). Waste Management during the COVID-19 Pandemic from Response to Recovery.

[135] Leja K., Lewandowicz G. (2010). Polymer biodegradation and biodegradable polymers-a review. Pol. J. Environ. Stud..

[136] Tebyetekerwa M., Xu Z., Yang S., Ramakrishna S. (2020). Electrospun nanofibers-based face masks. Adv. Fiber Mater..

[137] Nicosia A., Gieparda W., Foksowicz-Flaczyk J., Walentowska J., Wesołek D., Vazquez B., Prodi F., Belosi F. (2015). Air filtration and antimicrobial capabilities of electrospun PLA/PHB containing ionic liquid. Sep. Purif. Technol..

[138] Purwar R., Goutham K.S., Srivastava C.M. (2016). Electrospun Sericin/PVA/Clay nanofibrous mats for antimicrobial air filtration mask. Fibers Polym..

[139] Tiliket G., Le Sage D., Moules V., Rosa-Calatrava M., Lina B., Valleton J., Nguyen Q., Lebrun L. (2011). A new material for airborne virus filtration. Chem. Eng. J..

[140] De Sio L., Ding B., Focsan M., Kogermann K., Pascoal-Faria P., Petronela F., Mitchell G., Zussman E., Pierini F. (2021). Personalized Reusable Face Masks with Smart Nano-Assisted Destruction of Pathogens for COVID-19: A Visionary Road. Chem. Eur. J..

